# Sequential Thresholds Shape Drylands' Multitrophic Response to Aridification

**DOI:** 10.1111/ele.70242

**Published:** 2025-10-30

**Authors:** Jon Morant, José Antonio Sánchez‐Zapata, Marta Monfort‐Calatayud, Santiago Soliveres

**Affiliations:** ^1^ Department of Ecology University of Alicante Alicante Spain; ^2^ Departamento de Biología Aplicada Universidad Miguel Hernández Elche Spain; ^3^ Department of Applied Biology, Centro de Investigación e Innovación Agroalimentaria y Agroambiental (CIAGRO‐UMH) Miguel Hernández University of Elche, Avenida de la Universidad s/n Elche Spain; ^4^ Instituto Multidisciplinar para el Estudio del Medio–Ramon Margalef–Universidad de Alicante Alicante Spain

**Keywords:** anthropogenic disturbance, aridity thresholds, biodiversity loss, dryland ecosystems, primary productivity, trophic richness

## Abstract

Drylands, supporting significant global biodiversity, exhibit abrupt, non‐linear responses to increasing aridity with drastic declines after threshold crossing. While plant and soil responses are documented, impacts on other organisms remain unclear, as well as their potential interactions with other anthropogenic drivers. We investigated changes in taxonomic and trophic richness of multiple organisms, from bacteria to mammals, in response to aridity across 290 dryland ecoregions globally. All groups showed sequential threshold responses to aridity with threshold values ranging from 0.45 to 0.95 aridity levels, resulting in varying biodiversity losses (19%–54.3% depending on trophic group) after crossing such thresholds. Responses were most widespread in hyper‐arid and arid regions, primarily affecting herbivores and detritivores in semi‐arid areas, and were exacerbated by human disturbance and land‐use change. However, primary productivity and richness of primary producers and prior trophic levels partially buffered these declines. Biodiversity conservation and reducing anthropogenic pressures can mitigate these losses.

## Graphical abstract

We analyzed biodiversity responses across 290 global dryland ecoregions and found that all taxonomic and trophic groups, from microbes to mammals, showed abrupt threshold declines with increasing aridity. Thresholds varied (aridity 0.45–0.95), leading to 19%–54% biodiversity losses, most strongly in herbivores and detritivores and further exacerbated by land‐use change. While primary productivity and lower trophic richness provided some buffering, effective biodiversity conservation will require reducing anthropogenic pressures in drylands.

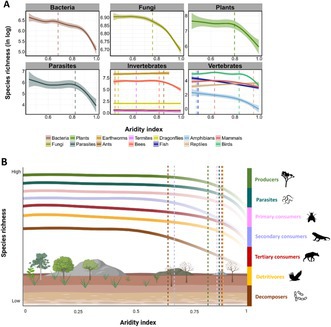

## 1 Introduction

Drylands cover approximately 45% of Earth's land surface, harbour 30%–40% of its living organisms and provide vital ecosystem services to a third of the global human population (Reynolds et al. 2007; Maestre et al. 2012). However, they are increasingly threatened by rising aridity, which may trigger abrupt changes in primary productivity, disruptions in plant–soil interactions and further plant biodiversity loss (Berdugo et al. 2020, 2022; Li et al. 2023). These changes contribute to desertification processes that threaten ecosystem integrity and human livelihoods globally (Reynolds et al. 2007). Since drylands are generally bottom‐up regulated (i.e., consumer‐driven by plant productivity; Schooley et al. 2018; Wagnon et al. 2024), these threshold responses to aridity observed in plants and soils likely scale up to higher trophic levels. However, the diverse organisms inhabiting drylands exhibit a range of physiological and behavioural adaptations that could partly mitigate the effects of increasing aridity (Maestre et al. 2016; Grünzweig et al. 2022). These potentially differential responses to aridity between primary producers and the organisms that interact with them (consumers, pollinators and detritivores) could induce decoupled dynamics (*sensu* Ochoa‐Hueso et al. 2021), likely impairing ecosystem functioning (Soliveres et al. 2016; Moi et al. 2021). Understanding responses to aridity across multiple taxa can aid in predicting the effects of increasing aridity on drylands' biodiversity and ecosystem functioning, and in developing strategies to enhance the resilience of these ecosystems under climate change scenarios.

In addition to aridification, drylands are particularly sensitive to and less protected from, land use changes and other anthropogenic disturbances (Lewin et al. 2024). These anthropogenic pressures could interact with the negative impacts of aridity on drylands, either buffering or amplifying these effects (Maestre et al. 2022a; Li et al. 2023; Suggitt et al. 2023; Montràs‐Janer et al. 2024). The effect of these disturbances may vary depending on the group of organisms, and the impact of these other global change factors on the diversity of drylands remains unclear. Intensive land uses and other anthropogenic pressures could reduce animal mobility or induce additional stressors, exacerbating negative effects of increasing aridity. However, wildlife can respond through physiological adaptations (torpor, osmoregulation), behavioural plasticity (temporal heat avoidance) and tolerance mechanisms (enhanced heat‐shock protein expression) (Stillman 2019; Albright et al. 2017). For less mobile taxa (amphibians, invertebrates), such adaptations may be critical for persistence during aridification. Conversely, human influence can enhance resource availability—artificial water points for livestock (Zhao et al. 2025) or increased plant productivity via irrigation, fertilisation, or nitrogen deposition—benefiting mobile and sessile species alike. Integrating responses across taxonomic groups with differing dispersal capacities and resilience traits enables better predictions of how biodiversity will cope with interacting climatic and anthropogenic pressures. Given the unique challenges faced by dryland ecosystems, including their susceptibility to degradation, it is crucial to understand the role of anthropogenic impacts as modulators of their response to ongoing climate change, to design effective strategies to mitigate the effects of aridification while preserving biodiversity and maintaining ecosystem functions in drylands.

Here, we studied the responses to increasing aridity of multiple dryland organisms, from bacteria to mammals, organized both taxonomically and by trophic group. We further evaluated how anthropogenic factors (i.e., human disturbance, climate change and land‐use change) and bottom‐up effects (i.e., mediated by plant richness and productivity, as well as the impact of the richness of previous trophic levels) modulated these responses to aridity. We hypothesize that aridity thresholds will vary amongst taxonomic groups and trophic levels due to divergent adaptive strategies. Soil bacteria and fungi maintain function under extreme dryness through dormancy or osmolyte synthesis (Schimel et al. 2007), whereas mobile vertebrates rely on behavioral thermoregulation or habitat tracking (Ward 2016). We predict multitrophic richness changes will vary amongst dryland ecoregions, as regional aridity gradients may interact with contrasting historical land‐use and biogeographic legacies (Huang et al. 2016). We expect producer richness and productivity to drive upper trophic‐level diversity via bottom‐up cascades in resource‐limited ecosystems (Borer et al. 2014). Conversely, anthropogenic pressures—habitat fragmentation (Newbold et al. 2015) and climate warming (Scheffers et al. 2016)—should exacerbate declines across all trophic levels by compressing thermal safety margins and disrupting interaction networks.

## 2 Methods

### 2.1 Data Acquisition

#### 2.1.1 Dryland Ecoregion Data

**FIGURE 1:**
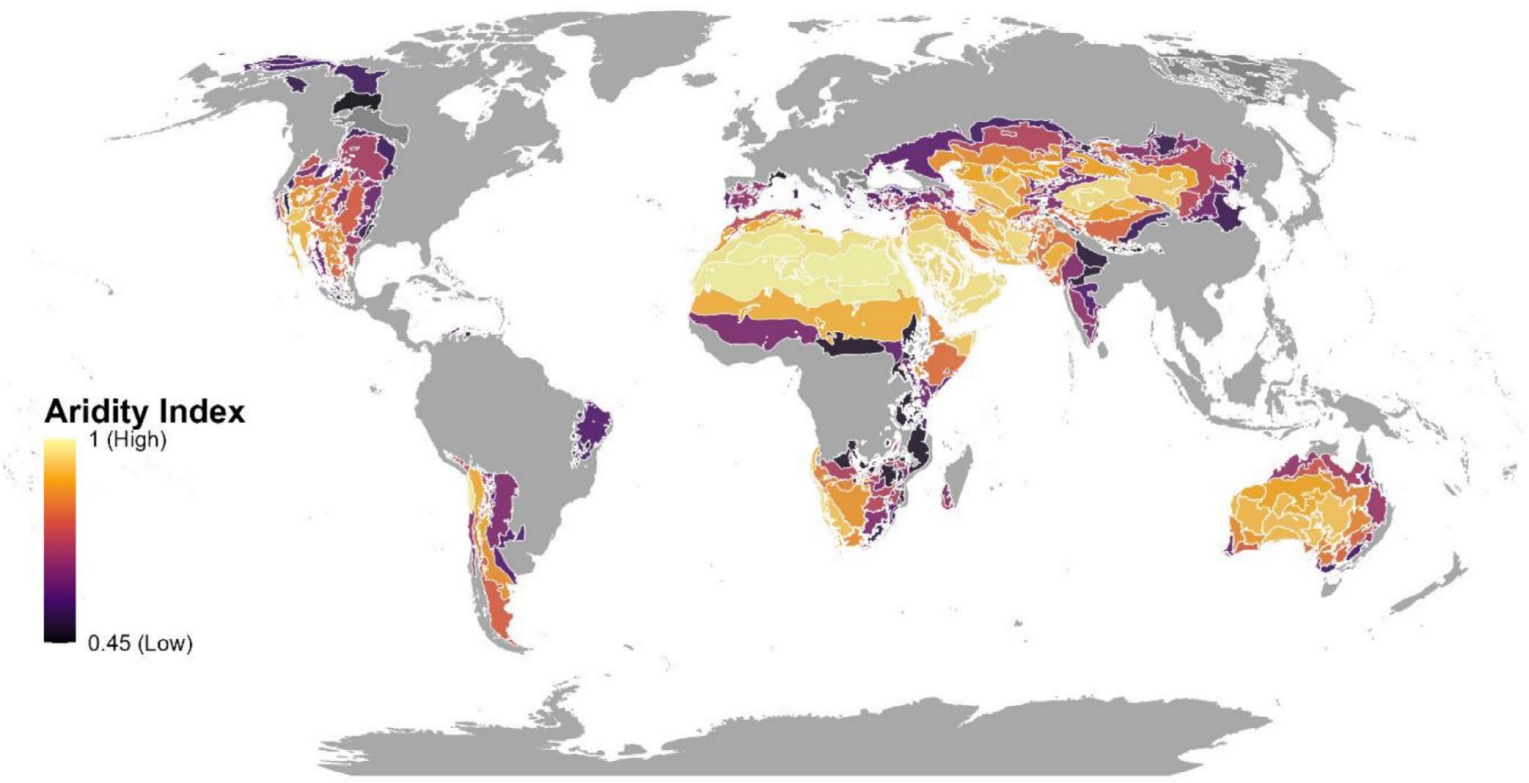
World drylands ecoregions (displayed in the Eckert IV equal‐area projection), delimited with white boundaries and their corresponding mean aridity values (1‐Aridity Index) (aridity values were extracted from Trabucco and Zomer 2019).

We used spatial data from dryland ecoregions as presented in Maestre et al. (2021), which builds on the baseline data from Dinerstein et al. (2017), widely used in global and regional conservation planning (Figure 1). We chose this dataset over the one provided by UNEP‐WCMC (United Nations Environment Programme World Conservation Monitoring Centre) because it offers a more detailed reflection of the biogeographical characteristics of each region, including vegetation types and community composition, in addition to aridity. Ecoregions offer an appropriate reference scale reflecting biome and habitat variations based on natural ecological processes. While delineated by biogeographic criteria independent of administrative boundaries, they provide a framework for assessing and managing natural and anthropogenic factors influencing biota (Yu et al. 2019). Given these considerations, we treat each dryland ecoregion (*n* = 290 dryland ecoregions) as a distinct sample unit for our analyses, allowing us to better capture the ecological complexity and biodiversity inherent in these regions. The mean aridity values within each dryland ecoregion were obtained from Trabucco and Zomer (2019).

#### 2.1.2 Species Richness Data and Grouping Criteria

We aimed to include as many taxa groups as possible to provide a comprehensive analysis of biodiversity responses across dryland ecoregions. We sourced species richness data from multiple origins, resolutions and temporal coverage (see Supporting Information Appendix S1 for details). Our final dataset includes one mean species richness value per taxon per dryland ecoregion, with data for bacteria, fungi, parasites, plants, earthworms, ants, termites, bees, dragonflies, fish, amphibians, reptiles, mammals and birds (Supporting Information Figure S1). Despite our efforts to gather a broad spectrum of taxa, we acknowledge that several groups—particularly insects and spiders—are underrepresented in our analyses due to the lack of spatially explicit richness data. Moreover, it should be mentioned that our ecoregion‐scale analysis may not reflect fine‐scale community dynamics within individual ecosystems, as our aim was to represent broad biogeographical patterns. We pooled these taxa into 6 major groups, including bacteria, fungi, parasites, plants, invertebrates (i.e., earthworms, ants, termites, bees and dragonflies) and vertebrates (i.e., fish, amphibians, reptiles, mammals and birds). We also grouped organisms according to their position in the food chain, adopting the classical one‐dimensional model of trophic dynamics proposed by Krebs (2009). Accordingly, we generated seven major trophic groups including producers (i.e., plants), parasites, primary consumers (i.e., invertebrates and vertebrate species known to consume or to include plants in their diet), secondary consumers (i.e., vertebrate species known to prey on the previous level), tertiary consumers (i.e., vertebrate species (i.e., predators) known to prey on the previous level), detritivores (i.e., vertebrate species known to consume carrion) and decomposers (i.e., bacteria and fungi) (see for instance Soliveres et al. 2016 for a similar approach) (see Supporting Information Appendix S2 for details).

#### 2.1.3 Anthropogenic Impact and Productivity Data

In addition to aridity, we gathered spatial data from three main anthropogenic impacts on dryland ecosystems, including human disturbance, temperature anomalies and land‐use change (Lewin et al. 2024; Osborne et al. 2022; García‐Vega and Newbold 2020). In the case of human disturbance, we carried out a fuzzy sum of multiple anthropogenic stressors known to have detrimental effects on ecosystems, namely: accessibility, road density, artificial light, livestock, population density and urbanisation (see Supporting Information Appendix S3 for details on layer sources and resolution). The fuzzy sum (hereafter FS) was based on the algebraic sum of a set of values between 0 and 1, which is given by FS = 1 − *∏*(1‐xi), where xi is each standardised layer of the set, such that the final fuzzy summed value is less than the literal sum of its parts, and tends towards a maximum value of 1 (Bonham‐Carter 2014).

To account for climate variability beyond average aridity trends, we used temperature anomaly as a climate diagnostic variable (climate change). Temperature anomalies represent deviations from long‐term reference periods and assess anthropogenic climate change effects (Jarvis and Forster 2024). We calculated mean temperature anomaly for each ecoregion over 1980–2022, using 1910–2000 average as baseline. Data were obtained at 2° resolution (200 km at equator) from Huang et al. (2024). We selected this variable because arid regions show greater sensitivity to temperature increases compared to humid regions (Sasaki et al. 2023). As for land‐use change, we selected change in frequency for cropland and pastures, respectively, between 1960 and 2019 with a resolution of 0.01 degrees (equivalent to 1 km at the equator) (see Winkler et al. 2021 for details). We chose these two parameters of change as they are regarded as the main land use across drylands worldwide and major desertification and global change drivers in drylands (Maestre et al. 2022a; Bestelmeyer et al. 2015). To assess the effect of ecosystem productivity, we selected NDVI (Normalised Difference Vegetation Index; hereafter NDVI) long‐term statistics between 1999 and 2019 from Copernicus at 1 km resolution (Copernicus 2024). NDVI provides information about net primary productivity, which is the energy basis of almost all ecosystems and the basis for the provision of many ecosystem services (Pettorelli et al. 2005).

Finally, for each dryland ecoregion, we obtained the mean aridity and mean richness values for each taxonomic group and trophic level, and of the above‐mentioned covariates (i.e., human disturbance, climate change, land‐use change and productivity).

### 2.2 Statistical Analyses

#### 2.2.1 Threshold Estimation

We performed threshold regression models to test aridity effects on species richness using the ‘chngpt’ package in R (Fong et al. 2017). These models detect non‐linear relationships by identifying thresholds where relationships fundamentally shift. We employed Gaussian error distribution with a ‘hinge’ model type, fitting continuous piecewise linear functions allowing slope changes at threshold points. This approach assumes normally distributed residuals, appropriate for continuous richness data, enabling standard linear model interpretation. Threshold regression permits both linear and non‐linear relationships, identifying critical points with abrupt response variable changes (e.g., sudden biodiversity increases/decreases). Models yield threshold values and bootstrapped 95% confidence intervals through maximum likelihood estimation and bootstrap resampling. We used aridity level (1—Aridity Index) as the primary covariate, representing water shortage and increasing dryness. Individual threshold models were performed for each taxonomic group (bacteria, fungi, parasites, invertebrates, vertebrates) and trophic level (primary producers, parasites, primary consumers, secondary consumers, tertiary consumers, detritivores, decomposers) separately to evaluate threshold points independently.

To assess threshold responses to aridity, we compared our threshold models with linear and null models using AIC and BIC (Berdugo et al. 2020). We checked spatial autocorrelation in model residuals using Moran's I tests to avoid bias in effect size estimates and significance from spatialized ecoregion data. Comprehensive model validation, including residual distribution and homoscedasticity assessments (Supporting Information), ensured result robustness and reliability.

#### 2.2.2 Identifying Areas of Multitrophic Richness Loss in Drylands

Using the analyses on aridity thresholds described above, we identified areas where multitrophic richness loss occurs in dryland ecoregions. To this end, we estimated which particular ecoregions have surpassed thresholds for each trophic level. We then estimated the percentage of area where species richness falls below thresholds for each trophic level within each dryland subtype (i.e., hyper‐arid, arid, semiarid and dry‐subhumid). We also estimated the mean species richness for each trophic level in areas above and below specific relative thresholds for that level, using spatial variation as a proxy for potential temporal changes (space‐for‐time substitution). Additionally, we calculated the percentage of richness loss for each trophic level to quantify the magnitude of biodiversity change.

#### 2.2.3 Multitrophic Richness Models

The threshold analyses allow only one predictor at a time (but see Gross et al. 2024 for alternatives). Since we were interested in identifying potential interactions between aridity and other anthropogenic and bottom‐up effects, and which specific drivers could buffer or multiply aridity effects, we performed complementary analyses for each group. To do so, we ran generalised linear models with a Gaussian distribution where we included species richness of each trophic level as the response variable and aridity level, human disturbance, temperature anomalies, change in pastures and change in croplands as covariates. To account for agricultural intensification effects on multitrophic richness patterns in dryland ecoregions, we included the interaction between pasture and cropland changes (land‐use change) to capture synergistic effects of agricultural intensification (or abandonment) on biodiversity patterns, as both changes likely co‐occur (Rey Benayas et al. 2007). Additionally, as we were interested in how each trophic level's richness modulates the immediate one's biodiversity patterns, we included the previous level's richness as a covariate. It should be noted that for higher trophic levels, the previous level richness was the result of summing the richness of all previous trophic levels. For instance, in the model for decomposers, the resulting covariate is the sum of the richness of parasites, primary consumers, secondary consumers, tertiary consumers and detritivores, since they benefit from other trophic levels. Moreover, since we expected that primary producers may have bottom‐up cascading effects on higher trophic‐level diversity, we added producers' richness (i.e., plant richness) and primary productivity (i.e., Normalised Difference Vegetation Index) as covariates in the models. All variables were scaled to provide comparable slope estimates. Prior to modelling, we computed Spearman's pairwise correlation between the covariates to evaluate potential multicollinearity in our models (Dormann et al. 2013). Accordingly, we did not remove any covariates since all of them showed correlation values <|0.7|(Supporting Information Figure S2).

We evaluated model performance by running ‘r2’ function from the ‘performance’ package (Lüdecke et al. 2021). We further estimated the variable importance by using the ‘varImp’ function from the ‘caret’ package (Kuhn 2008). We did not perform model simplification since we were interested in retaining all target covariate effects to compare across trophic levels. We estimated spatial correlation using Moran's I test and performed model diagnoses as in Section 2.2.1.

All spatial and statistical analyses were performed in R software version 4.4.0 (R Core Team 2024).

## 3 Results and Discussion

### 3.1 Aridity Thresholds Drive Taxonomic and Trophic Level Richness Patterns in Dryland Ecoregions

**FIGURE 2:**
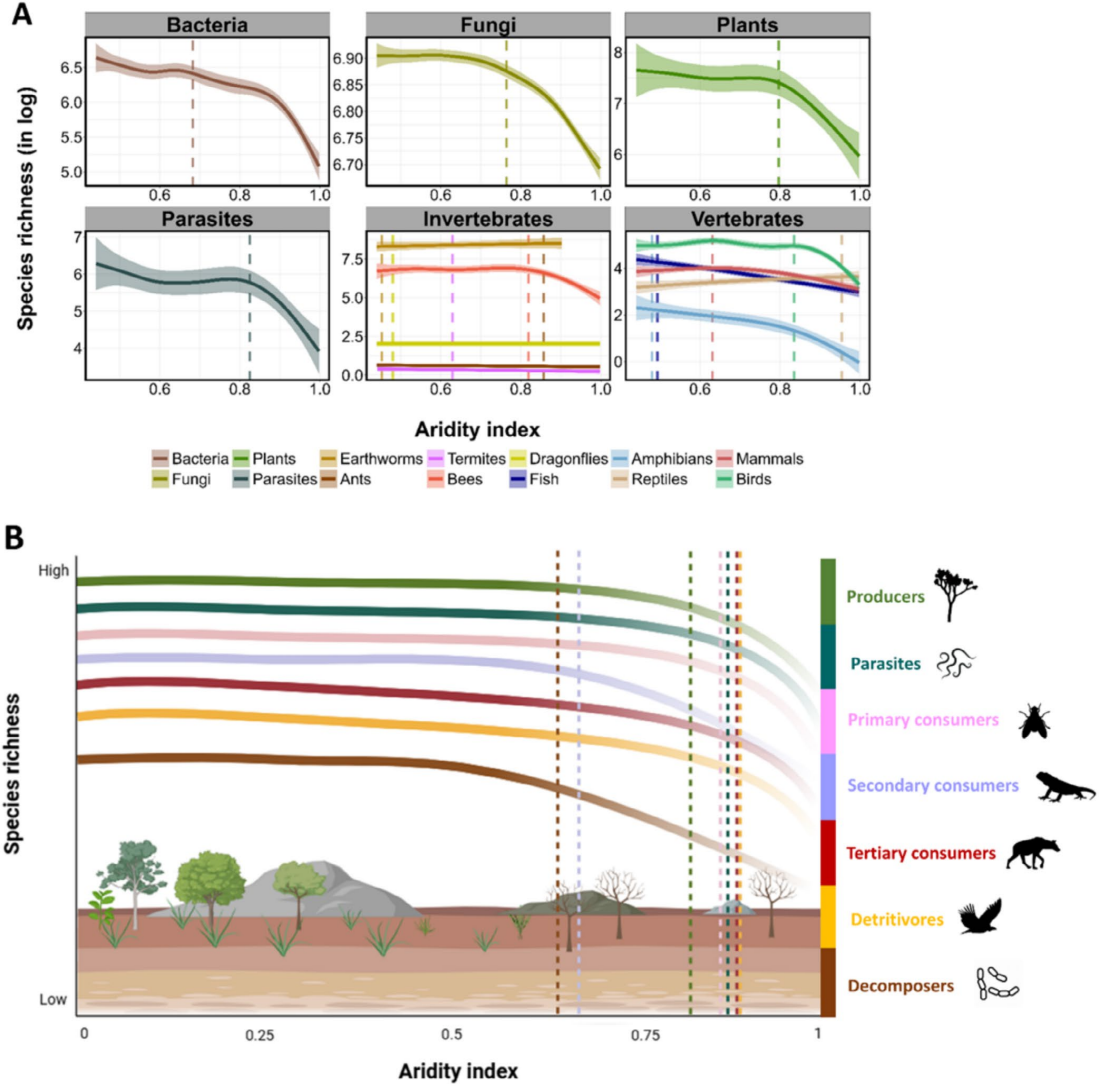
Aridity thresholds for each taxa (A) and trophic level (B). The dashed line represents the aridity threshold over which a change in richness occurs for each taxonomic group and trophic level, respectively. The background illustration for Figure B was generated using bioRender (https://www.biorender.com/). Silhouettes were obtained from PhyloPic (https://www.phylopic.org/). Note that the aridity gradient is reflected by 1‐AI, as it is more directly related to water shortage (higher values = drier sites). The lines are fitted using ‘gam’ method using ‘ggplot2’.

All organisms responded negatively to increasing aridity, showing sequential threshold responses (Figure 2A; see Supporting Information Tables S1–S3, Figure S3). First, organisms more dependent on water sources, such as fish, amphibians and dragonflies, showed earlier abrupt declines, accompanied by earthworms (~0.45), which require humid soils to thrive. The decline of these organisms was followed by bacteria, fungi, termites and mammals (~0.65), plants (0.79), bees, parasites and birds (~0.81) and finally reptiles (~0.95). These sequential abrupt declines in dryland biodiversity with aridity were consistent when organizing organisms in trophic levels (Figure 2B, Supporting Information Tables S1 and S4), and matched losses of 19%–54.3% of species, depending on the group (Supporting Information Table S5). Our findings extend the previously observed aridity thresholds in drylands' plants and soils (Berdugo et al. 2020) to include multiple groups of organisms largely varying in the movement ranges and physiological and behavioral adaptations. These results further support the higher sensitivity to aridification found in amphibians (Shi et al. 2021), as well as the stronger resistance of reptiles to such changes (Lewin et al. 2024).

### 3.2 Multitrophic Richness Loss in Dryland Ecoregions

**FIGURE 3:**
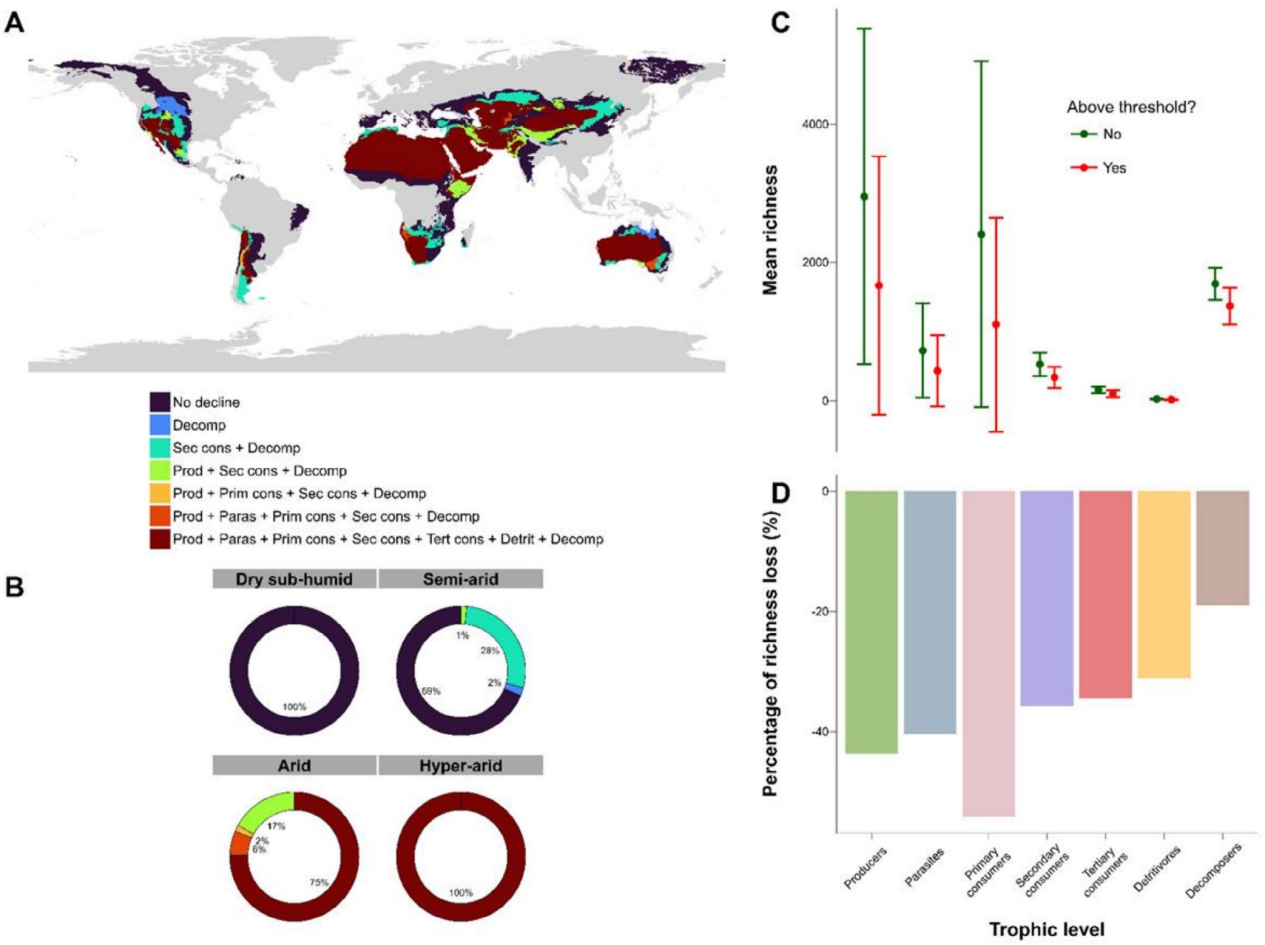
Multitrophic richness loss in drylands based on regions that crossed the aridity threshold for each trophic level (A), and percentage of area within dryland subtypes where multitrophic richness loss occurs (B). Overall (mean ± SE) richness (C), above/below the aridity threshold, as well as the percentage of change in species richness per trophic level once the aridity threshold is exceeded (D), are also shown. The grey areas for figure (A) represent non‐dryland regions. The abbreviations used in (A) are: Prod = producers, Paras = parasites, Prim cons = primary consumers, Sec cons = secondary consumers, Tert cons = tertiary consumers, Detrit = detritivores, Decomp = decomposers.

Multitrophic richness loss is not ubiquitous among the world's dryland ecoregions or dryland sub‐types (Figure 3A,B). Our findings indicate that while the decline in biodiversity is not widespread in semi‐arid regions (31% of such regions affected), they may experience a substantial thinning of intermediate trophic levels (most of the affected regions showed a disproportionately lower richness of secondary consumers and decomposers). This thinning of intermediate trophic levels could compromise fuel control and nutrient cycling in these areas (e.g., Leonard et al. 2010; Jing et al. 2015; Delgado‐Baquerizo et al. 2016). In general, the sequential thresholds observed across many dryland ecoregions, combined with contrasting proportions of biodiversity declines for different groups, could induce decoupling of crucial biotic interactions, impairing dryland ecosystem functioning (see Duffy 2002; Ochoa‐Hueso et al. 2021).

Other ecoregions were more sensitive than semi‐arid ones, with 75% of arid ecoregions and all hyper‐arid ones showing a more general decline across all trophic levels (Figure 3B). These results suggest that increased aridity leads to a widespread loss of multitrophic richness, potentially undermining the multifunctionality of dryland ecosystems. Given that drylands are predicted to expand by up to 23% in the coming decades (Huang et al. 2016) and that extreme climatic events like droughts and heatwaves are becoming more frequent and intense (Perkins‐Kirkpatrick and Lewis 2020; Ding et al. 2024), regions with lower aridity thresholds, such as semi‐arid and dry‐subhumid areas, may soon face severe impacts on ecosystem integrity, once they shift to drier dryland types. These effects, driven by multitrophic richness loss, are already seen in more arid and hyper‐arid regions.

### 3.3 Producers and Ecosystem Productivity Buffer Multitrophic Richness Loss in Drylands

**FIGURE 4:**
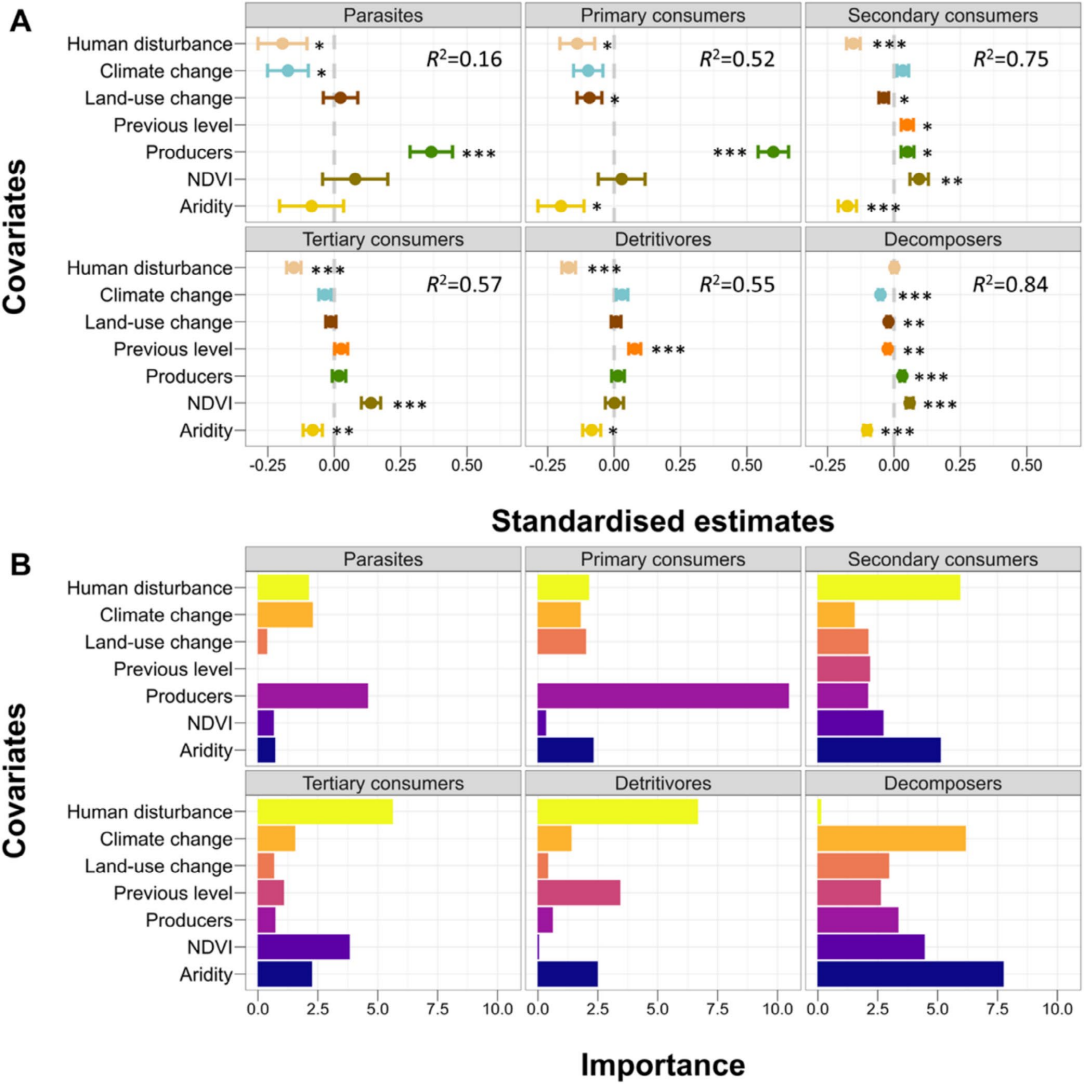
Standardised effects (A) and importance (B) of the biotic (productivity [NDVI] and plant richness [producers], previous trophic level richness), anthropogenic (human disturbance, land‐use changes) and climatic (aridity, temperature anomalies) drivers of multitrophic biodiversity patterns on each trophic level in drylands. The *R* ^*2*^ represents the variability explained by each model. The asterisks represent the significance of the covariates (*p* < 0.001***, *p* < 0.005**, *p* < 0.05*).

A general threshold response to aridity occurred across taxonomic groups, but sensitivities varied considerably. Primary consumers, producers and parasites suffered substantial species declines (> 40%; Figure 3C,D; Supporting Information Table S6), whereas parasites met their thresholds later—at higher aridity—and responded only weakly to aridity itself. Those groups less sensitive to aridity were highly responsive to plant productivity (decomposers, secondary and tertiary consumers), with positive relationships found in all cases. Whereas those more sensitive to aridity, did also respond strongly to plant diversity (parasites and primary consumers; Figure 4A,B). This bottom‐up modulation suggests that the sequential aridity thresholds we found are modulated by two different mechanisms of bottom‐up regulation, productivity and richness‐based. This modulating effect of bottom‐up mechanisms in drylands suggests that the expected multitrophic biodiversity declines could be potentially buffered in areas experiencing greening due to increased CO_2_ and changing rainfall patterns (Wang et al. 2022), or via restoration actions aimed at increasing productivity and plant richness in sensitive areas (see Li et al. 2022).

Responses to aridity in drylands' organisms can also be modulated by other anthropogenic pressures, such as increasing livestock grazing or land‐use changes (García‐Vega and Newbold 2020; Maestre et al. 2022b; Li et al. 2023; Lewin et al. 2024). We comprehensively evaluated the influence of these anthropogenic drivers by including shifting in cropland/rangeland cover (accounting for land‐use intensification and abandonment) and human disturbance (a compound index accounting for road and population density, urbanisation, light pollution or grazing intensity), as well as bottom‐up effects, mediated by plant richness and productivity and temperature anomalies (accounting for temporal climate variation measured through temperature anomalies in addition to average aridity changes). Our results demonstrated that both biotic (producers, previous‐level richness and ecosystem productivity) and environmental (temperature anomalies, human pressure and changes in cropland and grasslands) factors together with aridity modulate biodiversity change in drylands, overall accounting for 16%–84% of the variation observed in the richness across the different organisms studied (Figure 4A,B; Supporting Information Tables S7 and S8; Figure S4). Anthropogenic factors, including human disturbance and changes in croplands and grasslands, negatively affect richness patterns across all trophic levels in drylands. Drylands' biodiversity is highly sensitive to land‐use changes (García‐Vega and Newbold 2020), including land abandonment (Rey Benayas et al. 2007), a phenomenon further supported by the negative effects of land‐use change observed in three out of the six trophic groups evaluated. Furthermore, our results suggest that other anthropogenic pressures related to urbanisation, together with temperature anomalies and‐ most importantly‐bottom‐up control, are also strong drivers of dryland biodiversity change that need to be considered. In particular, and together with aridity, human disturbance was the most important negative driver of biodiversity change for secondary and tertiary consumers, as well as detritivores, highlighting habitat change, pollution, resource exploitation and invasive species as major disturbances to these organisms (Keck et al. 2025). Overall, anthropogenic pressures had either a negative or null effect on the biodiversity of different groups, which suggests that their role as buffering modulators of aridity is very unlikely in drylands. Indeed, our results suggest that reducing these pressures in those areas more sensitive to further aridification, such as those in the boundary between semi‐arid and arid, should be prioritised in order to mitigate biodiversity loss.

## 4 Conclusion

We showed that non‐linear responses to aridity in plants scale up to the multiple organisms inhabiting drylands, with the earliest aridity thresholds for those organisms associated with water and the latest for reptiles. Our results suggest that aridity thresholds interact with bottom‐up regulatory mechanisms, as evidenced by the strong effects of plant productivity and richness on higher trophic levels, associated with declines of species richness higher than 19% of current levels, and worsened by further anthropogenic disturbances and temperature anomalies. The aridity thresholds in multitrophic richness extend those previously reported (Berdugo et al. 2020) to include crucial groups in dryland conservation and functioning (e.g., mammals, reptiles, scavengers; Loft et al. 2024). Our study emphasizes the contrast between earlier responses to aridity and higher sensitivity (proportional richness changes), cautioning about potential decoupling in crucial biotic interactions that could compromise dryland ecosystem functioning. Our results reveal that biodiversity loss follows predictable sequential patterns, with primary producers declining first, followed by bottom‐up trophic cascades as aridity intensifies. In addition, anthropogenic pressures, including land‐use change, direct human disturbance and climate‐driven temperature anomalies, further exacerbate these losses, intensifying the speed and magnitude of biodiversity decline (Zhuang et al. 2024). According to our results, mitigation strategies should therefore prioritize reducing human impacts in the most vulnerable areas, as well as restoring plant richness and primary productivity, which have been shown to buffer against biodiversity loss and enhance ecosystem resistance to aridity. Such actions are critical for maintaining the ecological integrity and functioning of drylands, which cover nearly half of the world's terrestrial surface and are especially sensitive to ongoing global change.

## Author Contributions

**Jon Morant**, **José Antonio Sánchez‐Zapata** and **Santiago Soliveres:** conceptualisation. **Jon Morant**, **José Antonio Sánchez‐Zapata** and **Santiago Soliveres:** methodology. **Jon Morant:** investigation, visualisation, funding acquisition. **José Antonio Sánchez‐Zapata** and **Santiago Soliveres:** supervision. **Jon Morant:** writing – original draft. **Jon Morant**, **José Antonio Sánchez‐Zapata**, **Santiago Soliveres**, **Marta Monfort‐Calatayud:** writing – review and editing.

## Conflicts of Interest

The authors declare no conflicts of interest.

## Peer Review

The peer review history for this article is available at https://www.webofscience.com/api/gateway/wos/peer‐review/10.1111/ele.70242.

## Supporting information


**Data S1:** ele70242‐sup‐0001‐Supinfo.docx.

## Data Availability

The annotated dryland ecoregion dataset and R code to produce the analysis and figures are available at https://figshare.com/articles/dataset/Sequential_thresholds_shape_drylands_multitrophic_response_to_aridification/30231592. The dryland ecoregions map is available at https://zenodo.org/records/4252661. For data on species richness, (see Supporting Information Appendix S1). Productivity data (i.e., NDVI) are available at https://land.copernicus.eu/en/products/vegetation/normalised‐difference‐vegetation‐long‐term‐statistics‐v3‐0‐1km. The human disturbance layer is available upon request to the corresponding authors. The temperature anomalies layer was obtained from https://earthobservatory.nasa.gov/global‐maps/MOD_LSTAD_M. Land use change data are available at https://doi.pangaea.de/10.1594/PANGAEA.921846.
