# Supplementary material for: Sequential Thresholds Shape Drylands' Multitrophic Response to Aridification
Source: Ecol Lett. 2025 Oct 30;28(11):e70242. doi: 10.1111/ele.70242 (PMC12574969; doi:10.1111/ele.70242)
Supplement: Supplementary file 1 — Data S1: ele70242‐sup‐0001‐Supinfo.docx. [file ELE-28-0-s001.docx]

**SUPPLEMENTARY INFORMATION**

**Sequential thresholds shape drylands´ multitrophic response to aridification**

Jon Morant^1,*^, José Antonio Sánchez-Zapata^2,3^, Marta Monfort-Calatayud^1^, Santiago Soliveres^1,4^

1. Department of Ecology, University of Alicante, Alicante, Spain.
2. Departamento de Biología Aplicada, Universidad Miguel Hernández, Elche, Spain.
3. Department of Applied Biology, Centro de Investigación e Innovación Agroalimentaria y Agroambiental (CIAGRO-UMH), Miguel Hernández University of Elche, Avenida de la Universidad s/n, Elche, E-03202 Spain.
4. Instituto Multidisciplinar para el Estudio del Medio – Ramon Margalef – Universidad de Alicante, Alicante, Spain.

*Corresponding author: [jmorantetxebarria@gmail.com](mailto:jmorantetxebarria@gmail.com)

**Appendix 1**

Description of the species richness spatial layers for each taxonomic group selected, including bacteria, fungi, parasites, plants, ants, termites, bees, earthworms, dragonflies, freshwater fish, amphibians, mammals, and birds. For each target group, we provide source, resolution, and data treatment details. All species richness layers were reprojected to WGS84.

**Bacteria**

We obtained bacteria richness data from Delgado-Baquerizo, Oliverio et al. (2018) at 0.5 degrees resolution (equivalent to 50km at the equator). The map was built by using 237 soil samples from a global dataset (see Delgado-Baquerizo, Oliverio et al. 2018 for details). This dataset is freely available at <https://figshare.com/s/82a2d3f5d38ace925492> .

**Fungi**

We obtained fungi richness data from Mikryukov et al. (2023) at 0.0083 degrees resolution (equivalent to ~1km at the equator). The authors mapped global patterns of fungal taxonomic and phylogenetic alpha, beta, and gamma diversities based on long-read DNA sequencing of ca. 4000 topsoil samples collected during six global surveys, spanning 2010 to 2019, following comparable sampling and molecular analysis protocols (see Mikryukov et al. 2023 for details). The samples encompass a wide variety of land cover types, including different types of woody and herbaceous plant communities, deserts, and agricultural and urban environments.

**Plants**

For the plant richness, we used the data provided by Maestre et al. (2021) for each dryland ecoregion, which was computed as the number of species in the Global Biodiversity Information Facility Plantae dataset up to 2020 located in ecoregions with a mean aridity index lower than 0.65 (GBIF.org, [2020](https://nph.onlinelibrary.wiley.com/doi/10.1111/nph.17395#nph17395-bib-0052)).

**Parasites**

We collected parasite richness data from Carlson et al. (2020) at country resolution. This is, to our knowledge, the only map for this taxonomic group available. This dataset is the largest database of host–parasite associations and one of the world’s largest parasite collections up to and including 2020, accounting for 100,000–350,000 total species of helminth endoparasites of vertebrates, of which 85–95% are unknown to science. We rasterised all parasite richness maps (including known and undocumented species) at the country level for further purposes.

**Earthworms**

We gathered earthworm richness data from Phillips et al. (2019) at 0.745 degrees resolution (equivalent to ~70km at the equator). This dataset was created using data from 182 published and 17 unpublished papers between 1973 and 2017 (more than 7,000 sites), to compute global maps of the distribution of earthworm diversity, abundance, and biomass.

**Ants**

We collected ant richness data from Global Ant Biodiversity Informatics (GABI) Project (see Guénard et al. 2017 for details) at 0.865 degrees resolution (equivalent to ~80km at the equator). This dataset comprises occurrence data for valid ant species and subspecies data compiled as part of the Global Ant Biodiversity Informatics (GABI) Project, as of January 18, 2020.

**Termites**

We collated termite richness data from Liu et al. (2022) at 0.721 degrees resolution (equivalent to ~70km at the equator). This dataset was made up by collecting termite occurrences from datasets from Li et al., Liu et al., Cerezer et al., termite database records (https://www.termitediversity.org/) and GBIF records (https://doi.org/10.15468/dl.yd4edv), which included over 148151 records, almost entirely collated since 2000.

**Bees**

We collected pollinator richness data (referred to as 'bees' for consistency with the original dataset) from Bartomeus et al. (2019), at 0.891 degrees resolution (equivalent to ~80km at the equator). This dataset comprises species richness data for true bees (Apoidea, Hymenoptera) and hoverflies (Syrphidae, Diptera) from http://www.discoverlife.org, IUCN and http://www.syrphidae.com/ compiled primarily between 2007 and 2021. While these taxa belong to different orders, they were grouped based on their shared pollinator function. Species richness was calculated as the residuals of the log–log regression between pollinator species richness per country and country size. This correction accounts for the species–area relationship.

**Dragonflies**

We gathered data of dragonfly richness at a 0.5 degrees resolution (equivalent to ~50km at the equator) from the “rasterSP” package (rs-eco, 2023), who provide spatial information on distribution maps of dragonfly species from the IUCN updated to 2024. We generated such rasters manually by following instructions provided here <https://github.com/RS-eco/rasterSp> . For details on species range maps, please refer to IUCN main site <https://www.iucnredlist.org/> .

**Fish**

We obtained freshwater fish data from Atlas of Global Conservation (Hoekstra et al., University of California Press, 2010) with 0.982 degrees resolution (equivalent to ~90km at the equator). This map depicts freshwater fish species richness—the number of species present in each ecoregion—based on data compiled from various sources by Abell et al. (2008), reflecting species occurrences and knowledge available up to approximately 2010. Only species using freshwater for at least a portion of their life cycles, as identified using the habitat assignments in FishBase, are included. For the United States, NatureServe provided presence/absence data for individual species, coded to eight-digit hydrologic unit codes (HUCs); these HUC occurrences were then translated into ecoregions, and the data were manually cleaned of erroneous occurrences derived from species introductions and problematic records. For all other ecoregions, species lists were provided by experts based on published literature as well as from grey literature and unpublished sources. Abell et al. (2008) generated data on fish species for some small islands using FishBase and then augmented where possible with information from published literature. For a small number of ecoregions, it was impossible to generate species lists; therefore, richness estimates are provided instead. Extirpated species are included in these tallies, but confirmed extinct species, as determined by the Committee on Recently Extinct Organisms (data provided by Ian Harrison), and introduced and undescribed species are excluded.

**Amphibians, Mammals, Birds and Reptiles**

We gathered data of amphibians, mammals, birds and reptiles richness at a 0.5 degrees resolution (equivalent to ~50km at the equator) from the “rasterSP” package (rs-eco, 2023), who provide the most updated spatial information (~2023) on distribution maps of these groups from the IUCN. We generated such raster’s manually by following the instructions provided here https://github.com/RS-eco/rasterSp . As for the bird richness, data was obtained from BirdLife. We used the 2018 version, which comes in the form of individual shapefiles for each species. Reptile richness data were obtained from GARD reptile dataset and were downloaded from: <https://datadryad.org/resource/doi:10.5061/dryad.83s7k> (see Roll et al. 2017 for details).

**Appendix 2**

**Trophic level grouping criteria**

We classified species into seven major trophic level groups: producers, parasites, primary consumers, secondary consumers, tertiary consumers, detritivores, and decomposers, and estimated species richness for each group using a range of data sources. This classification was made based on dietary preferences and the trophic position of each group, with species assigned to trophic levels according to their predominant feeding strategy. When species occupied multiple trophic levels, they were classified based on their primary dietary component. Species-level dietary data were used when available; otherwise, we applied family-level generalizations while acknowledging potential classification uncertainty.

For producers, we used plant richness data from Maestre et al. (2021), which provided an estimate of plant species diversity across ecosystems. Parasite richness was estimated using data from Carlson et al. (2017), which compiled information on the diversity of parasitic species. For primary consumers, we estimated richness by aggregating data from various invertebrates, including earthworms, ants, termites, bees, and dragonflies, alongside herbivorous mammals and birds. Herbivorous species were selected from the PANTHERIA trait database (Jones et al. 2009) for mammals, focusing on predominantly plant-eating species within families such as Rodentia, Diprotodontia, Cetartiodactyla, Lagomorpha, Perissodactyla, and Proboscidea, and from the AVONET bird database (Tobias et al. 2022) for birds, focusing on herbivorous, granivorous, and frugivorous species from families such as Psittaciformes, Anseriformes, Columbiformes, Gruiformes, Struthioniformes, and Opisthocomiformes. For secondary consumers, we estimated richness by including insectivorous mammals and predatory birds that feed on primary consumers. The selected mammal families included those with predominantly insectivorous diets such as Chiroptera, Afrosoricida, Scandentia, and Eulipotyphla, while the bird families included those with primarily insectivorous or piscivorous diets. We also included fish, amphibians, and reptiles in this group, acknowledging the dietary diversity within these vertebrate groups. Tertiary consumers, which are apex predators that feed on secondary consumers, were represented by predominantly carnivorous species from mammal families such as Carnivora (excluding herbivorous and omnivorous species), Primates (carnivorous species), and Dasyuromorphia, and bird families such as Falconiformes, Strigiformes, Accipitriformes, Cathartiformes, Pelecaniformes, Ciconiiformes, Suliformes, Cariamiformes, and Podicipediformes. For detritivores, which feed on decomposing organic material, we estimated richness based on the work of Morant et al. (in prep) and Gutierrez-Cánovas et al. (2020), who gathered data from over 260 carrion-eating species across mammals, birds, and reptiles. Finally, for decomposers, we estimated richness by summing the diversity of bacteria and fungi species. This comprehensive approach integrated data from several authoritative sources to estimate species richness across all trophic levels, acknowledging that trophic classifications represent simplified generalizations of complex feeding relationships in natural ecosystems.

**Appendix 3**

**List and description of covariates used to develop the human disturbance layer**

Layers cover almost all terrestrial surfaces, and each layer measures the current extent of human modification of the natural environment. All layers were min-max bounded prior to their use in our algorithms, such that all values fell between 0 and 1, with 0 indicating the lowest level of modification and 1 indicating the highest level of modification. All layers were aggregated to 50km^2^ resolution. See the methods section for an explanation of the human disturbance layer generation.

A) Population density

We used the CIESIN Gridded Population of the World (GPW v4.11) human population density layer between 2000 and 2020 (CIESIN, 2018), in which the value for each cell is an estimate of number of persons per km2. We obtained the average value of population density per call between 2000-2020 period and aggregated this from a resolution of 2.5 arcminutes to 50 arcminutes (i.e., ~50km^2^).

B) Accessibility

We accessed gridded accessibility data openly available from Nelson et al. (2019). Accessibility data measures travel time (in hours) to every urban nucleus. Specifically, the dataset belongs to a suite of nine global travel-time accessibility indicators for the year 2015, at approximately one-kilometre spatial resolution, for a range of settlement size classes (see Nelson et al. 2019 for details). We obtained the averaged accessibility in hours from the nine layers to get a global mean value and aggregate our layer to 50 arcminute resolution (i.e., ~50km^2^). We rescaled this variable so that values with 0 (lower threat) were those pixels showing largest travelling time whereas we classified as 1 (higher threat value) cells with smaller travelling time to urban nuclei.

C) Road density

We obtained road density measures as meters of road per km^2^ from GLOBIO for 2005-2014 years (Meijer et al. 2018). We used all road type density including major highways, primary, secondary, tertiary and local roads at 5 arcminute resolution and aggregated to 50 arcsecond resolution (i.e., ~50km^2^).

D) Livestock density

We extracted livestock density from Gridded Livestock of the World (GLW; Robinson et al. 2014). GLW is now in its fourth version and is a peer-reviewed spatial dataset on the global distribution and abundance of livestock species for 2015 and includes global distributions of cattle, buffaloes, sheep, goats, horses, pigs, chickens and ducks at a spatial resolution of 5 arcminutes, approximately 10 km at the equator. We summed all livestock type densities to get a unique value of livestock density per cell and aggregated data to 50 arcminute resolution (i.e., ~50km^2^).

E) Power lines

We obtained predicted data for all low, medium and high voltage infrastructures in kilometres per cell at global level from Aderne et al. (2020), which summarised all power lines kilometres per cell at global to get a unique value per cell. We aggregated this value to 50 arcminutes resolution (i.e., ~50km^2^).

F) Urbanization

We used the 250-m resolution Global Human Settlement built-up areas layer (Corbane et al., 2018) for the year 2014 (layer ID: GHS_BUILT_LDS2014_GLOBE_R2018A_54009_250_V2_0), which represent built-up areas (expressed as probabilities). We reprojected to EPSG:4236 at a resolution of 50 arcminutes (i.e., ~50km^2^).

G) Artificial light

We used global artificial light raster generated by Falchi et al. (2016) which measures artificial component of the night sky brightness measured in simulated zenith radiance (μcd/m^2^) at 1km resolution. We aggregated the raster to 50km^2^ resolution.

**Tables**

**Table S1**. Model selection criteria (AIC, Delta_AIC) comparing Null, Linear, and chngpt (i.e., changepoint) models for each group within taxonomic and trophic categories. The “Best” column indicates the model with the lowest AIC. The results show how well each model fits species richness data across groups, helping to identify whether a threshold response to aridity is supported.

| **Group_Category** | **Group** | **Model** | **AIC** | **Delta_AIC** | **Best** |
| --- | --- | --- | --- | --- | --- |
| Vertebrate | Amphibians | Changepoint | 2034.30989 | 0 | TRUE |
| Invertebrate | Ants | Changepoint | 1139.254151 | 0.419963817 | FALSE |
| Taxonomic | Bacteria | Changepoint | 3898.867753 | 0 | TRUE |
| Invertebrate | Bees | Changepoint | 4746.795003 | 0 | TRUE |
| Vertebrate | Birds | Changepoint | 3292.508671 | 0 | TRUE |
| Trophic | Decomposers | Changepoint | 3942.57387 | 0 | TRUE |
| Vertebrate | Fish | Changepoint | 2977.785671 | 0 | TRUE |
| Taxonomic | Fungi | Changepoint | 2984.720659 | 0 | TRUE |
| Taxonomic | Invertebrates | Changepoint | 17580.33093 | 0 | TRUE |
| Vertebrate | Mammals | Changepoint | 2728.980263 | 0 | TRUE |
| Invertebrate | Odonates | Changepoint | 2679.198991 | 0 | TRUE |
| Taxonomic | Parasites | Changepoint | 4487.297882 | 0 | TRUE |
| Taxonomic | Plants | Changepoint | 5277.043848 | 0 | TRUE |
| Trophic | Primary_Consumers | Changepoint | 5277.778784 | 0 | TRUE |
| Vertebrate | Reptiles | Changepoint | 2745.365314 | 0 | TRUE |
| Trophic | Scavengers | Changepoint | 2024.811367 | 0 | TRUE |
| Trophic | Secondary_Consumers_Ter | Changepoint | 3717.961421 | 0 | TRUE |
| Invertebrate | Termites | Changepoint | 564.601685 | 0 | TRUE |
| Trophic | Tertiary_Consumers | Changepoint | 3043.621314 | 0 | TRUE |
| Taxonomic | Vertebrates | Changepoint | 16133.72153 | 0 | TRUE |
| Invertebrate | Worms | Changepoint | 683.3112021 | 1.10380699 | FALSE |
| Vertebrate | Amphibians | Linear | 2035.501326 | 1.191435608 | FALSE |
| Invertebrate | Ants | Linear | 1139.880476 | 1.046288696 | FALSE |
| Taxonomic | Bacteria | Linear | 3906.281452 | 7.413698525 | FALSE |
| Invertebrate | Bees | Linear | 4759.678167 | 12.88316384 | FALSE |
| Vertebrate | Birds | Linear | 3310.546515 | 18.03784392 | FALSE |
| Trophic | Decomposers | Linear | 3962.806163 | 20.23229309 | FALSE |
| Vertebrate | Fish | Linear | 2978.958359 | 1.172687994 | FALSE |
| Taxonomic | Fungi | Linear | 3071.795255 | 87.07459674 | FALSE |
| Taxonomic | Invertebrates | Linear | 17586.76731 | 6.436381301 | FALSE |
| Vertebrate | Mammals | Linear | 2737.806844 | 8.826580791 | FALSE |
| Invertebrate | Odonates | Linear | 2679.641227 | 0.442236053 | FALSE |
| Taxonomic | Parasites | Linear | 4494.559805 | 7.261922608 | FALSE |
| Taxonomic | Plants | Linear | 5292.188636 | 15.14478791 | FALSE |
| Trophic | Primary_Consumers | Linear | 5286.996889 | 9.218105574 | FALSE |
| Vertebrate | Reptiles | Linear | 2749.670868 | 4.305553405 | FALSE |
| Trophic | Scavengers | Linear | 2060.444194 | 35.63282684 | FALSE |
| Trophic | Secondary_Consumers_Ter | Linear | 3745.954367 | 27.99294688 | FALSE |
| Invertebrate | Termites | Linear | 566.0827173 | 1.481032252 | FALSE |
| Trophic | Tertiary_Consumers | Linear | 3080.176822 | 36.55550785 | FALSE |
| Taxonomic | Vertebrates | Linear | 16141.15652 | 7.434987544 | FALSE |
| Invertebrate | Worms | Linear | 683.2663435 | 1.0589484 | FALSE |
| Vertebrate | Amphibians | Null | 2106.731898 | 72.42200744 | FALSE |
| Invertebrate | Ants | Null | 1138.834187 | 0 | TRUE |
| Taxonomic | Bacteria | Null | 4022.207976 | 123.3402231 | FALSE |
| Invertebrate | Bees | Null | 4773.370416 | 26.57541257 | FALSE |
| Vertebrate | Birds | Null | 3377.606289 | 85.09761809 | FALSE |
| Trophic | Decomposers | Null | 4125.660507 | 183.0866368 | FALSE |
| Vertebrate | Fish | Null | 3035.239934 | 57.45426324 | FALSE |
| Taxonomic | Fungi | Null | 3293.94657 | 309.225911 | FALSE |
| Taxonomic | Invertebrates | Null | 17599.0671 | 18.73617623 | FALSE |
| Vertebrate | Mammals | Null | 2790.436388 | 61.45612498 | FALSE |
| Invertebrate | Odonates | Null | 2685.638982 | 6.439991246 | FALSE |
| Taxonomic | Parasites | Null | 4507.984522 | 20.68664006 | FALSE |
| Taxonomic | Plants | Null | 5313.064264 | 36.02041621 | FALSE |
| Trophic | Primary_Consumers | Null | 5308.178255 | 30.39947161 | FALSE |
| Vertebrate | Reptiles | Null | 2748.077523 | 2.712209266 | FALSE |
| Trophic | Scavengers | Null | 2106.799068 | 81.98770123 | FALSE |
| Trophic | Secondary_Consumers_Ter | Null | 3876.357263 | 158.3958428 | FALSE |
| Invertebrate | Termites | Null | 565.2341324 | 0.632447341 | FALSE |
| Trophic | Tertiary_Consumers | Null | 3157.136039 | 113.5147254 | FALSE |
| Taxonomic | Vertebrates | Null | 16213.50281 | 79.78128643 | FALSE |
| Invertebrate | Worms | Null | 682.2073951 | 0 | TRUE |

**Table S2**. Results of the aridity threshold models for each taxonomic group and trophic level species richness. For each model set, model results and the yielded threshold value for aridity are shown. Note that “(Aridity-chngpt)+” corresponds to 𝜷1 in equation (hinge). In other words, it is the change in slope as the covariate richness crosses the threshold. Abbreviations: SE= Standard Error, CI=Confidence Interval. Significant terms and highlighted in bold.

| **Taxonomic group** | **Terms** | **Coefficients** | **SE** | **Low 95% CI** | **Upper 95 % CI** | **p-value** |
| --- | --- | --- | --- | --- | --- | --- |
| Bacteria | Intercept | 691.8884 | 44.29129 | 636.5454 | 810.1673 | **<0.001** |
|  | (Aridity-chngpt)+ | -1349.5864 | 531.74961 | -2931.7557 | -847.2972 | **<0.001** |
|  |  | **Value** | **SE** | **Low 95% CI** | **Upper 95 % CI** |  |
|  | Threshold: | 0.68255732 | 0.09239411 | 0.47267029 | 0.8348552 |  |
| Fungi | Intercept | 993.2019 | 3.220734 | 987.9961 | 1000.6214 | **<0.001** |
|  | (Aridity-chngpt)+ | -755.2644 | 98.710926 | -927.0296 | -540.0828 | **0.039** |
|  |  | **Value** | **SE** | **Low 95% CI** | **Upper 95 % CI** |  |
|  | Threshold: | 0.7644663 | 0.02399349 | 0.70211272 | 0.7961672 |  |
| Parasites | Intercept | 723.8407 | 82.94577 | 616.719 | 941.8665 | **<0.001** |
|  | (Aridity-chngpt)+ | -3420.4773 | 2848.62568 | -12297.698 | -1131.0855 | 0.229 |
|  |  | **Value** | **SE** | **Low 95% CI** | **Upper 95 % CI** |  |
|  | Threshold: | 0.8245882 | 0.1164402 | 0.4811214 | 0.9375670 |  |
| Plants | Intercept | 2964.258 | 199.749 | 2626.133 | 3409.151 | **<0.001** |
|  | (Aridity-chngpt)+ | -12815.582 | 2512.861 | -18334.853 | -8484.436 | **<0.001** |
|  |  | **Value** | **SE** | **Low 95% CI** | **Upper 95 % CI** |  |
|  | Threshold: | 0.7965648 | 0.0292366 | 0.7373489 | 0.8519563 |  |
| Earthworms | Intercept | 4800.342 | 685.4367 | 3472.501 | 6159.413 | **<0.001** |
|  | (Aridity-chngpt)+ | 2684.656 | 14850.8948 | -48250.512 | 9964.996 | 0.857 |
|  |  | **Value** | **SE** | **Low 95% CI** | **Upper 95 % CI** |  |
|  | Threshold: | 0.4525386 | 0.1052177 | 0.4405916 | 0.8530452 |  |
| Ants | Intercept | 2.273792 | 0.2044768 | 1.95787 | 2.75942 | **<0.001** |
|  | (Aridity-chngpt)+ | -3.762996 | 21.2494636 | -9.239235 | 74.05866 | 0.859 |
|  |  | **Value** | **SE** | **Low 95% CI** | **Upper 95 % CI** |  |
|  | Threshold: | 0.8569302 | 0.1260601 | 0.4811214 | 0.975277 |  |
| Termites | Intercept | 1.945599 | 0.2399494 | 1.537591 | 2.478193 | **<0.001** |
|  | (Aridity-chngpt)+ | -2.130463 | 3.1074627 | -13.807284 | -1.62603 | 0.490 |
|  |  | **Value** | **SE** | **Low 95% CI** | **Upper 95 % CI** |  |
|  | Threshold: | 0.62946654 | 0.09644263 | 0.51578125 | 0.89383637 |  |
| Bees | Intercept | 1343.791 | 89.78802 | 1163.586 | 1515.555 | **<0.001** |
|  | (Aridity-chngpt)+ | -5850.469 | 4365.91306 | -20834.52 | -3720.141 | 0.180 |
|  |  | **Value** | **SE** | **Low 95% CI** | **Upper 95 % CI** |  |
|  | Threshold: | 0.8196266 | 0.04727542 | 0.75224739 | 0.93756702 |  |
| Dragonflies | Intercept | 23.83776 | 3.699837 | 16.44056 | 30.94392 | **<0.001** |
|  | (Aridity-chngpt)+ | -26.17498 | 11.647678 | -58.31585 | -12.65695 | **0.024** |
|  |  | **Value** | **SE** | **Low 95% CI** | **Upper 95 % CI** |  |
|  | Threshold: | 0.48021151 | 0.09815743 | 0.47267029 | 0.857 |  |
| Fish | Intercept | 87.72868 | 7.385699 | 70.95493 | 99.906 | **<0.001** |
|  | (Aridity-chngpt)+ | -133.55165 | 18.168637 | -175.41972 | -104.198 | **<0.001** |
|  |  | **Value** | **SE** | **Low 95% CI** | **Upper 95 % CI** |  |
|  | Threshold: | 0.49357969 | 0.03952009 | 0.47454781 | 0.629 |  |
| Amphibians | Intercept | 15.62371 | 1.70093 | 11.51635 | 18.183 | **<0.001** |
|  | (Aridity-chngpt)+ | -27.10034 | 4.255689 | -39.85113 | -23.168 | **<0.001** |
|  |  | **Value** | **SE** | **Low 95% CI** | **Upper 95 % CI** |  |
|  | Threshold: | 0.480 | 0.056 | 0.467 | 0.689 |  |
| Reptiles | Intercept | 43.427 | 3.015 | 35.306 | 47.126 | **<0.001** |
|  | (Aridity-chngpt)+ | -476.481 | 269.939 | -1025.869 | 32.293 | 0.075 |
|  |  | **Value** | **SE** | **Low 95% CI** | **Upper 95 % CI** |  |
|  | Threshold: | 0.954 | 0.113 | 0.529 | 0.973 |  |
| Birds | Intercept | 174.056 | 11.428 | 162.882 | 207.683 | **<0.001** |
|  | (Aridity-chngpt)+ | -874.042 | 204.288 | -1102.823 | -302.012 | **<0.001** |
|  |  | **Value** | **SE** | **Low 95% CI** | **Upper 95 % CI** |  |
|  | Threshold: | 0.8348552 | 0.062 | 0.616 | 0.862 |  |
| Mammals | Intercept | 64.941 | 4.915 | 56.670 | 75.941 | **<0.001** |
|  | (Aridity-chngpt)+ | -102.727 | 33.632 | -213.713 | -81.874 | **0.002** |
|  |  | **Value** | **SE** | **Low 95% CI** | **Upper 95 % CI** |  |
|  | Threshold: | 0.630 | 0.087 | 0.478 | 0.819 |  |
| **Trophic level** |  |  |  |  |  |  |
| Primary consumers | Intercept | 2383.69 | 284.3564 | 2047.517 | 3162.194 | **<0.001** |
|  | (Aridity-chngpt)+ | -13821.72 | 3142.5905 | -16827.797 | -4508.842 | **<0.001** |
|  |  | **Value** | **SE** | **Low 95% CI** | **Upper 95 % CI** |  |
|  | Threshold: | 0.819 | 0.087 | 0.485 | 0.827 |  |
| Secondary consumers | Intercept | 538.916 | 21.307 | 498.504 | 582.029 | **<0.001** |
|  | (Aridity-chngpt)+ | -1262.385 | 294.424 | -2116.888 | -962.745 | **<0.001** |
|  |  | **Value** | **SE** | **Low 95% CI** | **Upper 95 % CI** |  |
|  | Threshold: | 0.706 | 0.04630371 | 0.628 | 0.809 |  |
| Tertiary consumers | Intercept | 155.146 | 5.525 | 148.805 | 170.463 | **<0.001** |
|  | (Aridity-chngpt)+ | -672.578 | 149.602 | -849.813 | -263.370 | **<0.001** |
|  |  | **Value** | **SE** | **Low 95% CI** | **Upper 95 % CI** |  |
|  | Threshold: | 0.834 | 0.049 | 0.663 | 0.857 |  |
| Detritivores | Intercept | 24.595 | 0.751 | 23.288 | 26.244 | **<0.001** |
|  | (Aridity-chngpt)+ | -98.060 | 19.510 | -123.108 | -46.626 | **<0.001** |
|  |  | **Value** | **SE** | **Low 95% CI** | **Upper 95 % CI** |  |
|  | Threshold: | 0.837 | 0.035 | 0.721 | 0.862 |  |
| Decomposers | Intercept | 1687.732 | 31.312 | 1624.801 | 1747.546 | **<0.001** |
|  | (Aridity-chngpt)+ | -1971.935 | 516.003 | -3447.381 | -1424.647 | **<0.001** |
|  |  | **Value** | **SE** | **Low 95% CI** | **Upper 95 % CI** |  |
|  | Threshold: | 0.696 | 0.048 | 0.623 | 0.812 |  |

**Table S3.** Moran's I test results for spatial autocorrelation in model residuals across taxonomic and trophic groups for aridity threshold models. The table shows the Moran's I statistic (observed spatial autocorrelation), expected value under spatial randomness, variance, p-value, sample size (n), and group labels for each trophic group. Negative values suggest dispersion, while positive values indicate clustering of similar residuals.

| **Group** | **Moran_I** | **Expected** | **p_value** |
| --- | --- | --- | --- |
| Plants | 0.019 | 0.019 | < 2e-16 |
| Parasites | 0.013 | 0.013 | 3.97E-11 |
| Bacteria | -0.008 | -0.008 | 0.95515 |
| Fungi | 0.018 | 0.018 | < 2e-16 |
| Worms | -0.018 | -0.018 | 0.36484 |
| Ants | -0.006 | -0.006 | 0.76574 |
| Termites | -0.015 | -0.015 | 0.95182 |
| Bees | 0.125 | 0.125 | < 2e-16 |
| Odonates | -0.017 | -0.017 | 1 |
| Fish | 0.011 | 0.011 | 2.32E-08 |
| Amphibians | 0.013 | 0.013 | 3.57E-11 |
| Reptiles | 0.009 | 0.009 | 9.36E-07 |
| Mammals | 0.027 | 0.027 | < 2e-16 |
| Birds | 0.015 | 0.015 | 8.91E-14 |
| Primary_Consumers | 0.024 | 0.024 | < 2e-16 |
| Secondary_Consumers | 0.003 | 0.003 | 0.00422 |
| Tertiary_Consumers | 0.001 | 0.001 | 0.02759 |
| Detritivores | -0.005 | -0.005 | 0.75175 |
| Decomposers | -0.005 | -0.005 | 0.72686 |

**Table S4.** Aridity threshold response by taxonomic group. Aridity threshold values are given as 1-Aridity index (unitless), together with Standard Error and the Lower and Upper 95% Confidence Intervals.

| **Name** | **Taxonomic group** | **Aridity threshold ± SE** | **Lower-Upper 95% CI** |
| --- | --- | --- | --- |
| Bacteria | Bacteria | 0.682 ± 0.092 | 0.472 - 0.834 |
| Fungi | Fungi | 0.764 ± 0.023 | 0.702 - 0.796 |
| Plants | Plants | 0.796 ± 0.029 | 0.737 - 0.851 |
| Parasites | Parasites | 0.824 ± 0.116 | 0.481 - 0.937 |
| Earthworms | Invertebrates | 0.452 ± 0.105 | 0.441 - 0.853 |
| Ants | Invertebrates | 0.856 ± 0.126 | 0.481 - 0.975 |
| Termites | Invertebrates | 0.629 ± 0.096 | 0.515 - 0.893 |
| Bees | Invertebrates | 0.819 ± 0.047 | 0.752 - 0.937 |
| Dragonflies | Invertebrates | 0.480 ± 0.098 | 0.472 - 0.857 |
| Fish | Vertebrates | 0.493 ± 0.039 | 0.474 - 0.629 |
| Amphibians | Vertebrates | 0.480 ± 0.056 | 0.467 - 0.689 |
| Reptiles | Vertebrates | 0.954 ± 0.113 | 0.529 - 0.973 |
| Mammals | Vertebrates | 0.630 ± 0.087 | 0.478 - 0.819 |
| Birds | Vertebrates | 0.834 ± 0.062 | 0.616 - 0.862 |

**Table S5.** Aridity threshold response by trophic level. Aridity threshold values are given as 1-Aridity index (unitless), together with Standard Error and the Lower and Upper 95% Confidence Intervals.

| **Trophic level** | **Aridity threshold ± SE** | **Upper-Lower 95% CI** |
| --- | --- | --- |
| Producers | 0.796 ± 0.029 | 0.737 - 0.851 |
| Parasites | 0.824 ± 0.116 | 0.481 - 0.937 |
| Primary consumers | 0.819 ± 0.087 | 0.485 - 0.827 |
| Secondary consumers | 0.706 ± 0.046 | 0.628 - 0.809 |
| Tertiary consumers | 0.834 ± 0.049 | 0.663 - 0.857 |
| Detritivores | 0.837 ± 0.035 | 0.721 - 0.862 |
| Decomposers | 0.696 ± 0.048 | 0.623 - 0.812 |

**Table S6.** Mean richness and percentage of richness loss per trophic level above and after crossing the aridity thresholds obtained from threshold models. SD=Standard deviation from mean richness.

| **Trophic level** | **Above threshold?** | **Mean richness** | **SD** | **Percentage of change in richness** |
| --- | --- | --- | --- | --- |
| Producers | No | 2955 | 2424 | -43.7 |
|  | Yes | 1664 | 1870 |  |
| Primary consumers | No | 2410 | 2501 | -54.3 |
|  | Yes | 1102 | 1548 |  |
| Secondary consumers | No | 526 | 171 | -35.8 |
|  | Yes | 338 | 155 |  |
| Tertiary consumers | No | 155 | 48 | -34.5 |
|  | Yes | 102 | 51.5 |  |
| Detritivores | No | 24.6 | 8.92 | -31.1 |
|  | Yes | 17 | 7.17 |  |
| Decomposers | No | 1689 | 229 | -19 |
|  | Yes | 1368 | 264 |  |
| Parasites | No | 724 | 680 | -40.4 |
|  | Yes | 432 | 515 |  |

**Table S7.** Results of the negative binomial models for multitrophic richness loss. Abbreviations: SE=Standard Error.

| **Trophic level** |  | **Estimate** | **SE** | **z value** | **P value** | **Variable Importance** |
| --- | --- | --- | --- | --- | --- | --- |
| Parasites | Intercept | 6.275 | 0.071 | 87.844 | **<0.001** |  |
|  | Producers’ richness | 0.332 | 0.078 | 4.252 | **<0.001** | 4.252 |
|  | NDVI | 0.084 | 0.094 | 0.893 | 0.372 | 0.893 |
|  | Land-use change | 0.275 | 0.088 | 3.121 | **0.002** | 3.121 |
|  | Climate change | -0.227 | 0.074 | -3.079 | **0.002** | 3.079 |
|  | Human disturbances | -0.300 | 0.074 | -4.029 | **<0.001** | 4.029 |
| Primary consumers | Intercept | 7.348 | 0.052 | 141.499 | **<0.001** |  |
|  | Producers’ richness | 0.530 | 0.057 | 9.351 | **<0.001** | 9.351 |
|  | NDVI | 0.171 | 0.068 | 2.527 | **0.012** | 2.527 |
|  | Land-use change | 0.265 | 0.064 | 4.126 | **<0.001** | 4.126 |
|  | Climate change | -0.106 | 0.054 | -1.968 | **0.049** | 1.968 |
|  | Human disturbances | -0.222 | 0.053 | -4.164 | **<0.001** | 4.164 |
| Secondary consumers | Intercept | 6.040 | 0.022 | 279.587 | **<0.001** |  |
|  | Previous level richness | 0.063 | 0.024 | 2.554 | **0.011** | 2.554 |
|  | Producers’ richness | 0.048 | 0.025 | 1.882 | **0.060** | 1.882 |
|  | NDVI | 0.203 | 0.028 | 7.190 | **<0.001** | 7.190 |
|  | Land-use change | 0.000 | 0.027 | 0.009 | 0.993 | 0.009 |
|  | Climate change | 0.018 | 0.022 | 0.802 | 0.422 | 0.802 |
|  | Human disturbances | -0.238 | 0.022 | -10.668 | **<0.001** | 10.668 |
| Tertiary consumers | Intercept | 4.907 | 0.022 | 227.136 | **<0.001** |  |
|  | Previous level richness | 0.039 | 0.025 | 1.566 | 0.117 | 1.566 |
|  | Producers’ richness | 0.019 | 0.025 | 0.759 | 0.448 | 0.759 |
|  | NDVI | 0.192 | 0.028 | 6.789 | **<0.001** | 6.789 |
|  | Land-use change | -0.044 | 0.027 | -1.629 | 0.103 | 1.629 |
|  | Climate change | -0.037 | 0.022 | -1.645 | 0.1 | 1.645 |
|  | Human disturbances | -0.190 | 0.022 | -8.503 | **<0.001** | 8.503 |
| Detritivores | Intercept | 3.059 | 0.020 | 149.964 | **<0.001** |  |
|  | Previous level richness | 0.074 | 0.023 | 3.295 | **0.001** | 3.295 |
|  | Producers’ richness | 0.014 | 0.024 | 0.582 | 0.561 | 0.582 |
|  | NDVI | 0.047 | 0.027 | 1.749 | 0.080 | 1.749 |
|  | Land-use change | 0.072 | 0.024 | 2.988 | **0.003** | 2.988 |
|  | Climate change | 0.026 | 0.021 | 1.247 | 0.212 | 1.247 |
|  | Human disturbances | -0.207 | 0.021 | -9.852 | **<0.001** | 9.852 |
| Decomposers | Intercept | 7.313 | 0.009 | 831.453 | **<0.001** |  |
|  | Previous level richness | -0.013 | 0.010 | -1.298 | 0.194 | 1.298 |
|  | Producers’ richness | 0.029 | 0.010 | 2.787 | **0.005** | 2.787 |
|  | NDVI | 0.124 | 0.012 | 10.699 | **<0.001** | 10.699 |
|  | Land-use change | -0.005 | 0.011 | -0.478 | 0.632 | 0.478 |
|  | Climate change | -0.056 | 0.009 | -6.083 | **<0.001** | 6.083 |
|  | Human disturbances | -0.044 | 0.009 | -4.732 | **<0.001** | 4.732 |

**Table S8.** Moran's I test results for spatial autocorrelation in model residuals across trophic groups. The table shows the Moran's I statistic (observed spatial autocorrelation), expected value under spatial randomness, variance, p-value, sample size (n), and group labels for each trophic group. Negative values suggest dispersion, while positive values indicate clustering of similar residuals.

| **Moran_I** | **Expected** | **p_value** | **Group** |
| --- | --- | --- | --- |
| 0.055 | -0.004 | <0.001 | Parasites |
| 0.039 | -0.004 | <0.001 | Primary_consumers |
| 0.018 | -0.004 | <0.001 | Secondary_consumers |
| -0.012 | -0.004 | 0.994 | Tertiary_consumers |
| -0.019 | -0.004 | 1.000 | Detritivores |
| -0.004 | -0.004 | 0.511 | Decomposers |


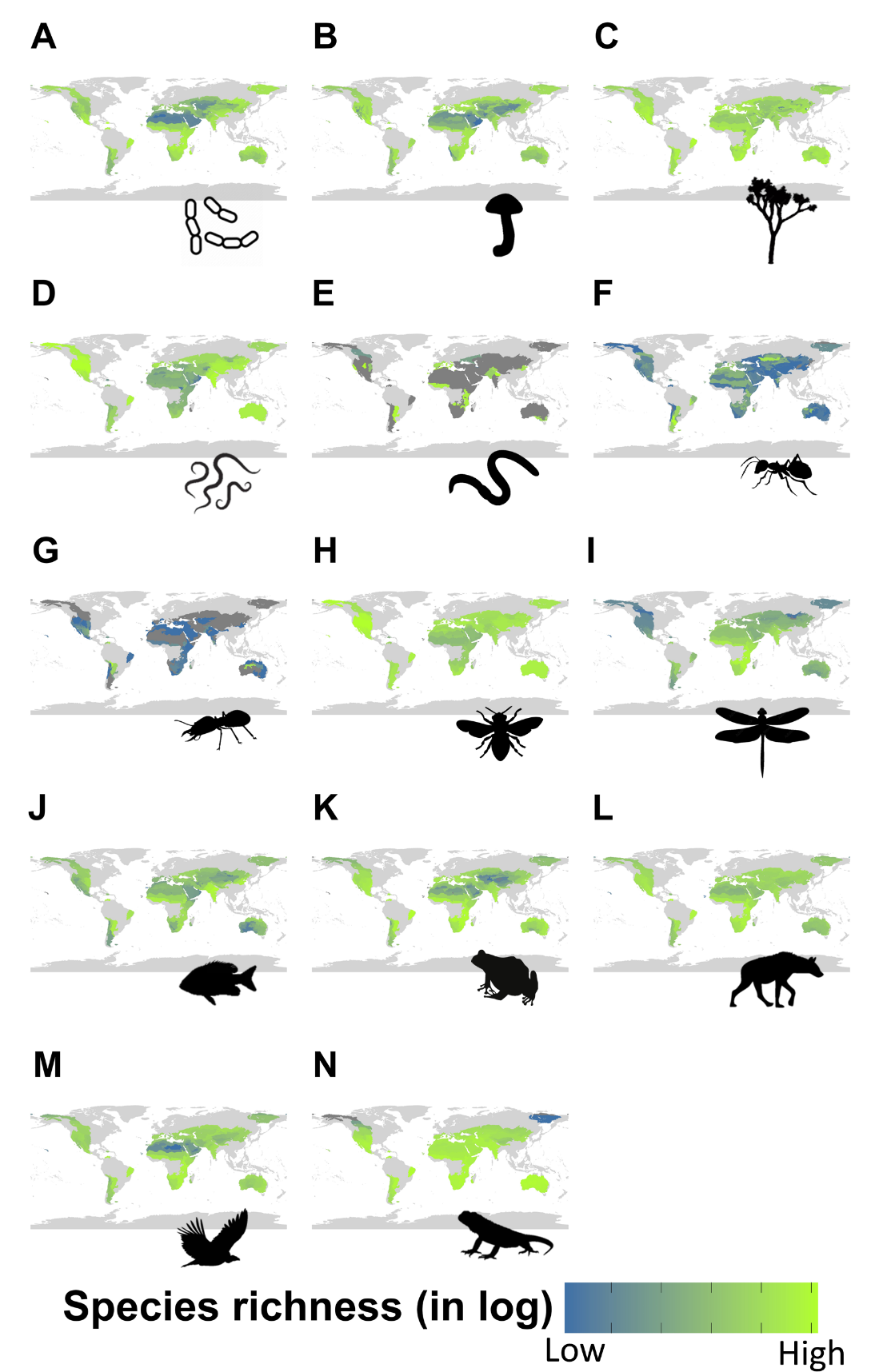


**Fig. S1.** Mean species richness (in logarithmic scale) in dryland ecoregions for each major organismal group, including bacteria (A), fungi (B), plants (C), parasites (D), earthworms (e), ants (F), termites (G), bees (H), dragonflies (I), fish (J), amphibians (K), mammals (L), birds (M), and reptiles (N). Please, refer to the methods section for sources and calculations of mean species richness. Categorical (Low and High) labels have been used in this figure for the sake of simplicity.

**
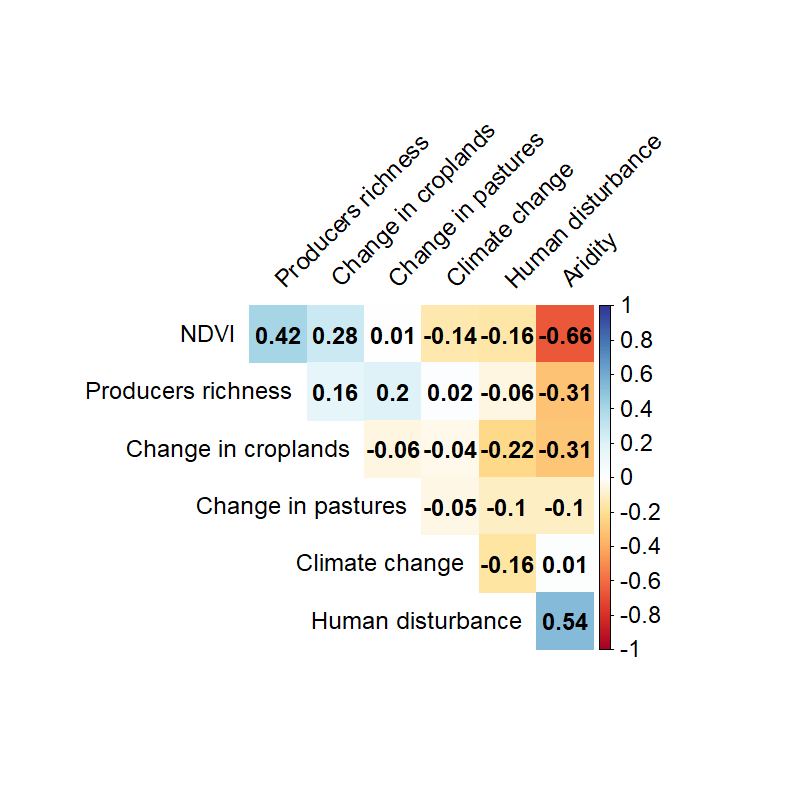
**

**Fig. S2.** Paired Spearman correlation matrix of the covariates included in the multitrophic level richness models.

**
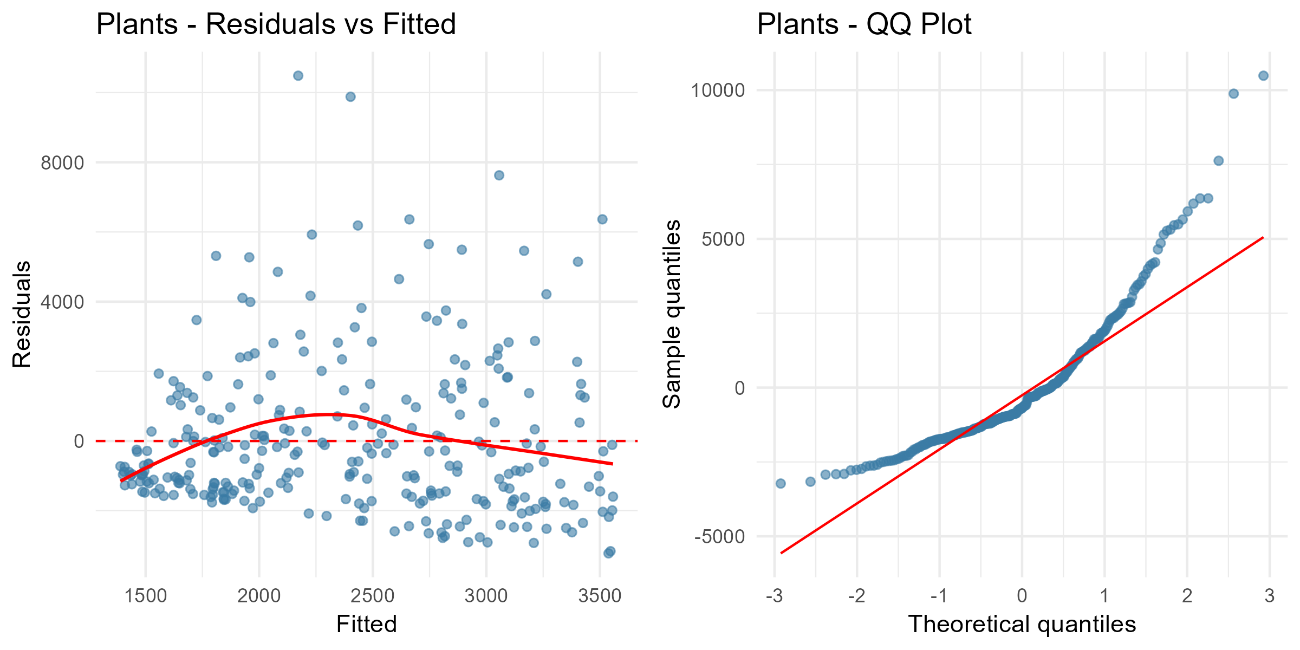
**

**
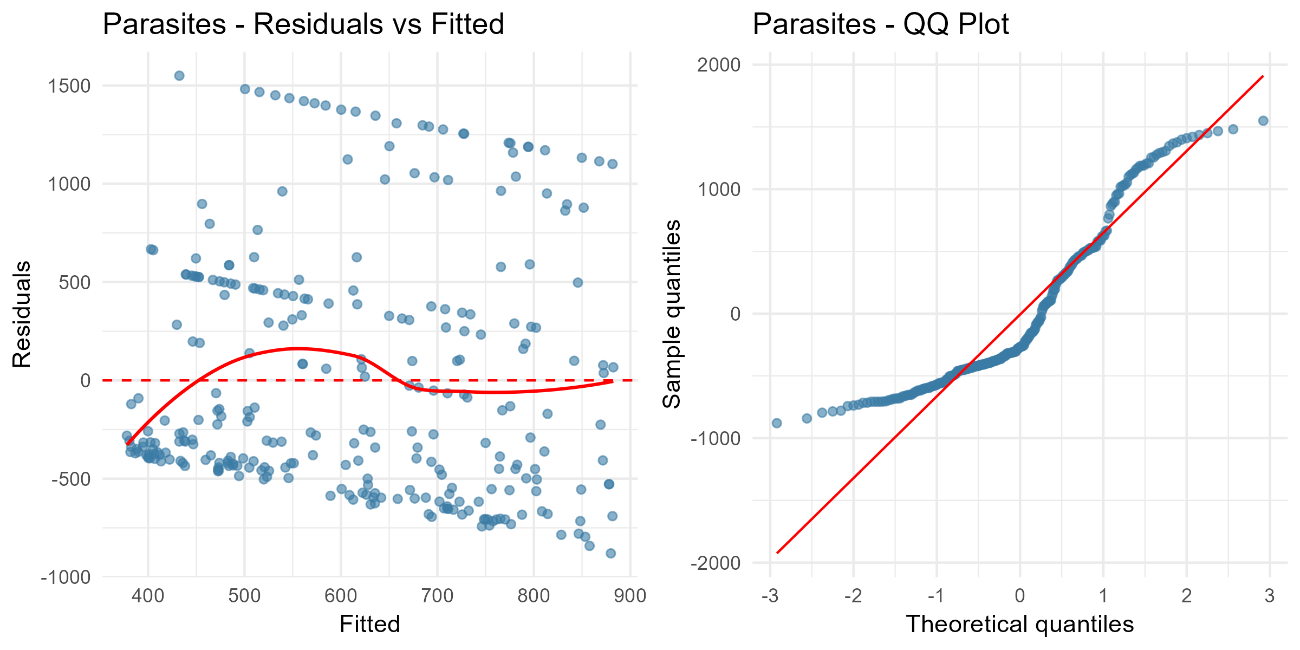
**

**
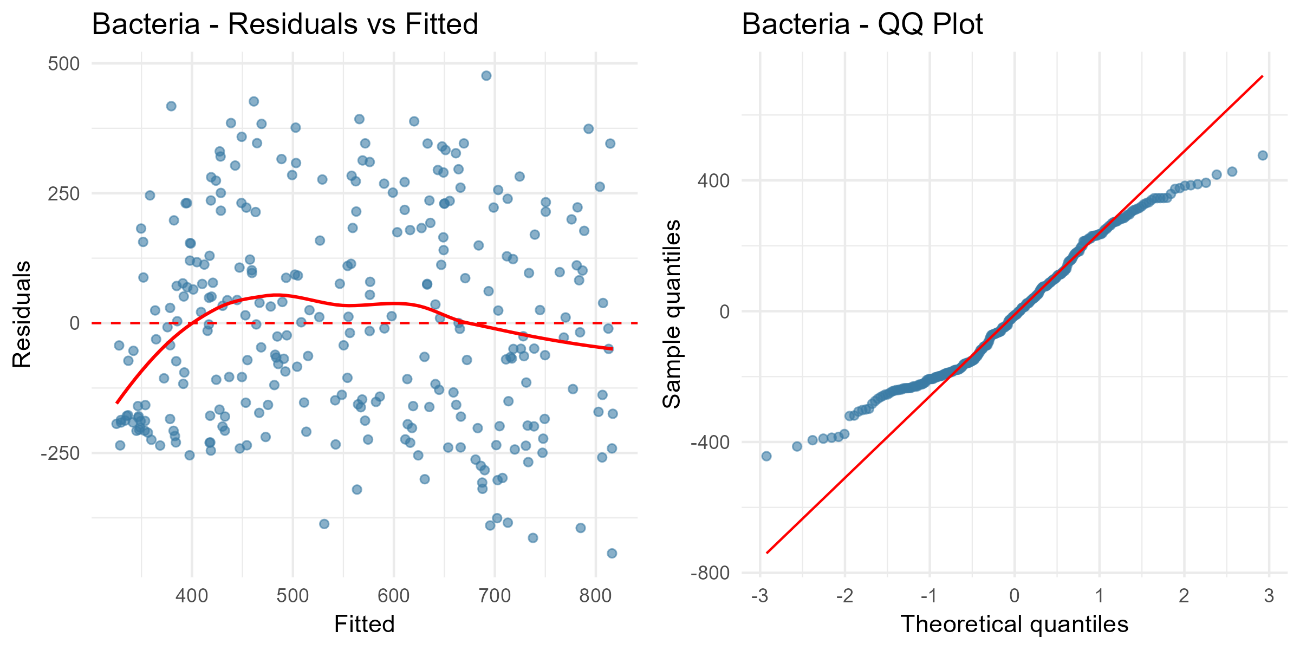
**

**
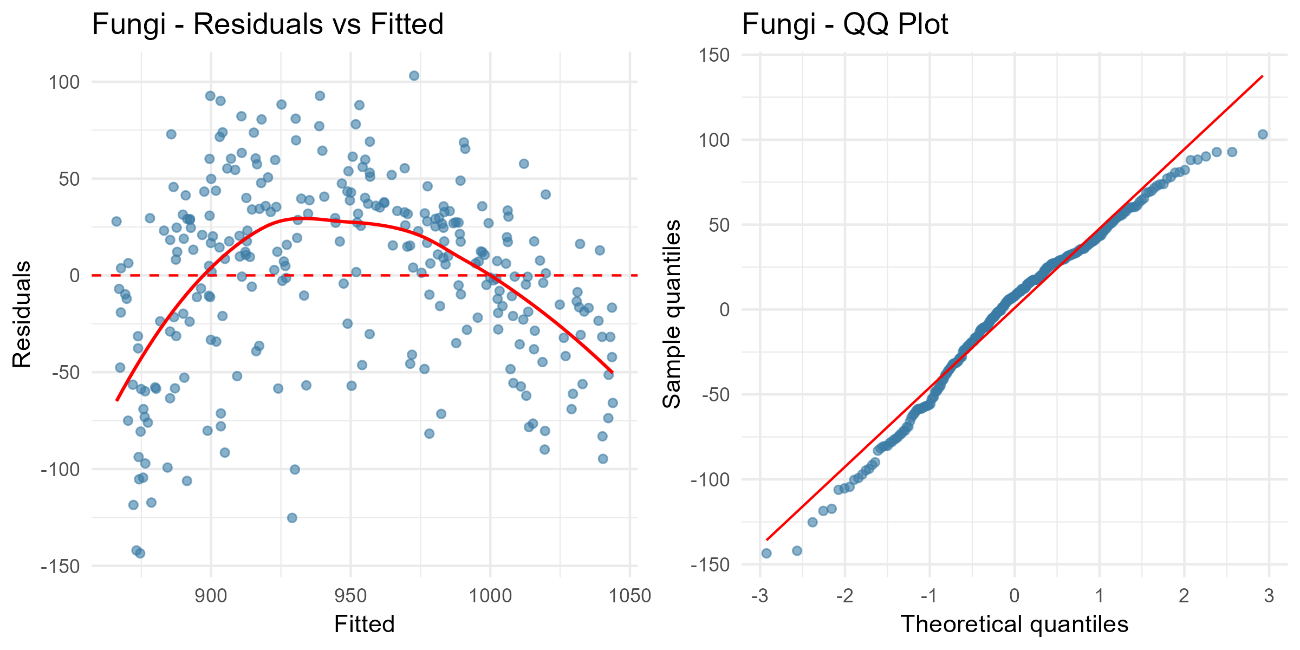
**

**
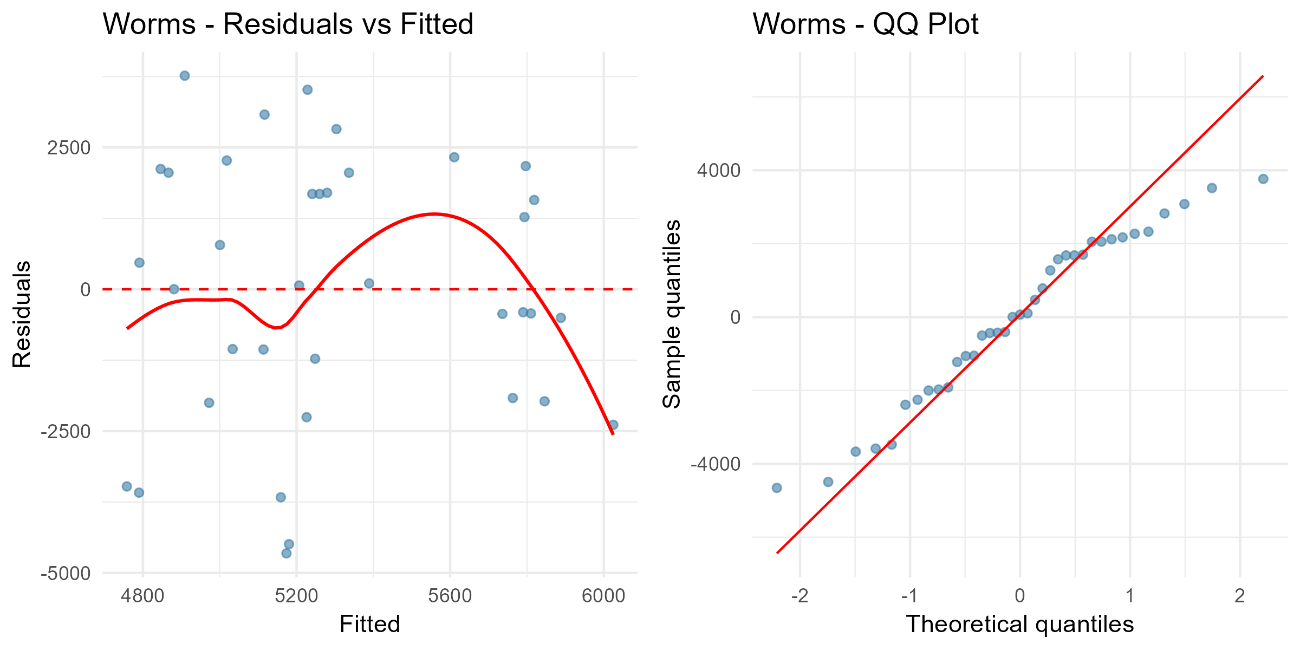
**

**
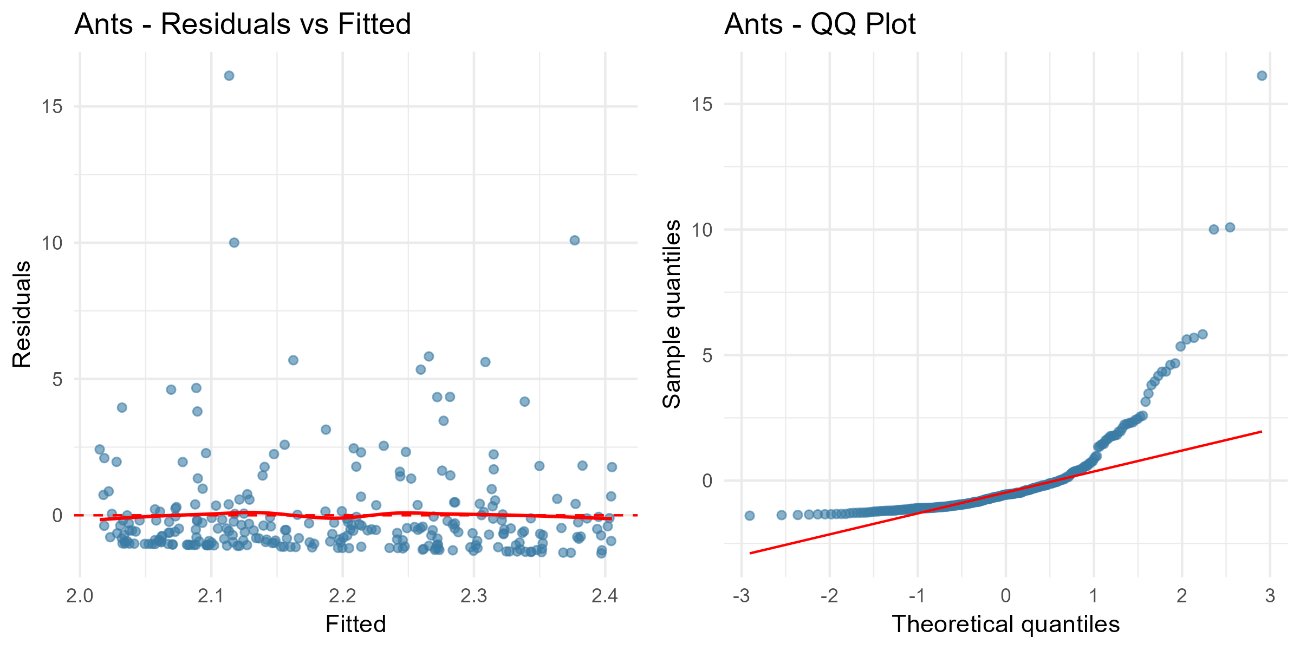
**

**
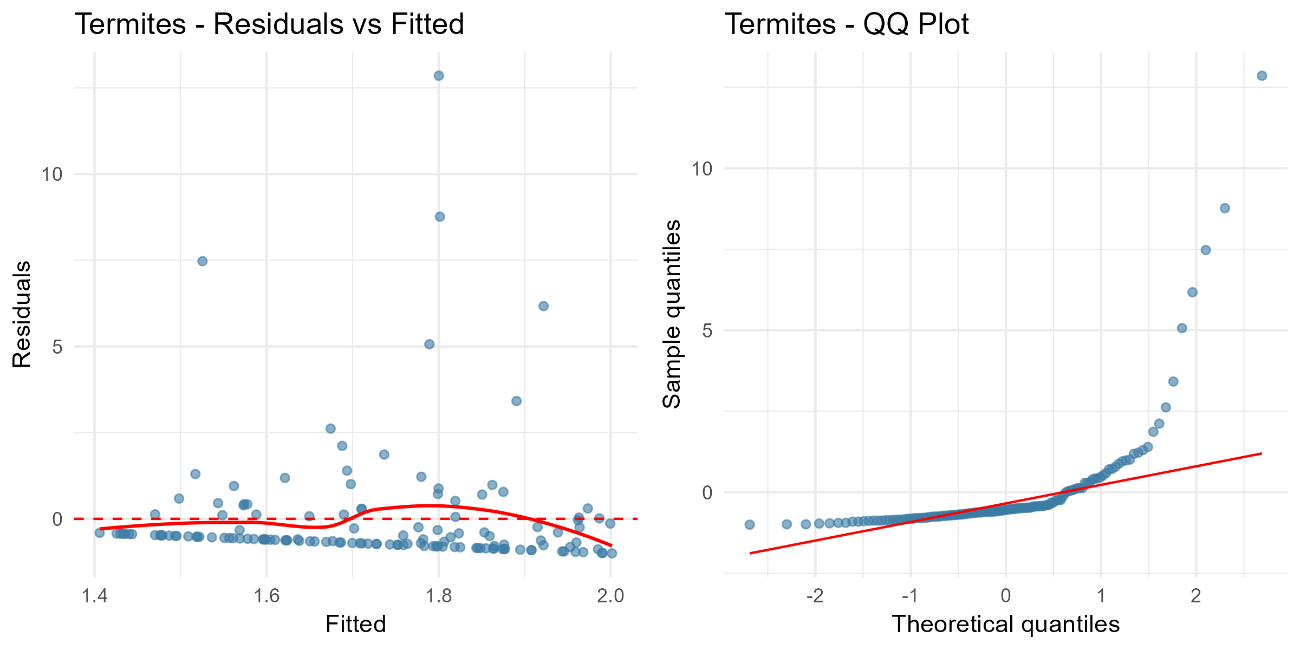
**

**
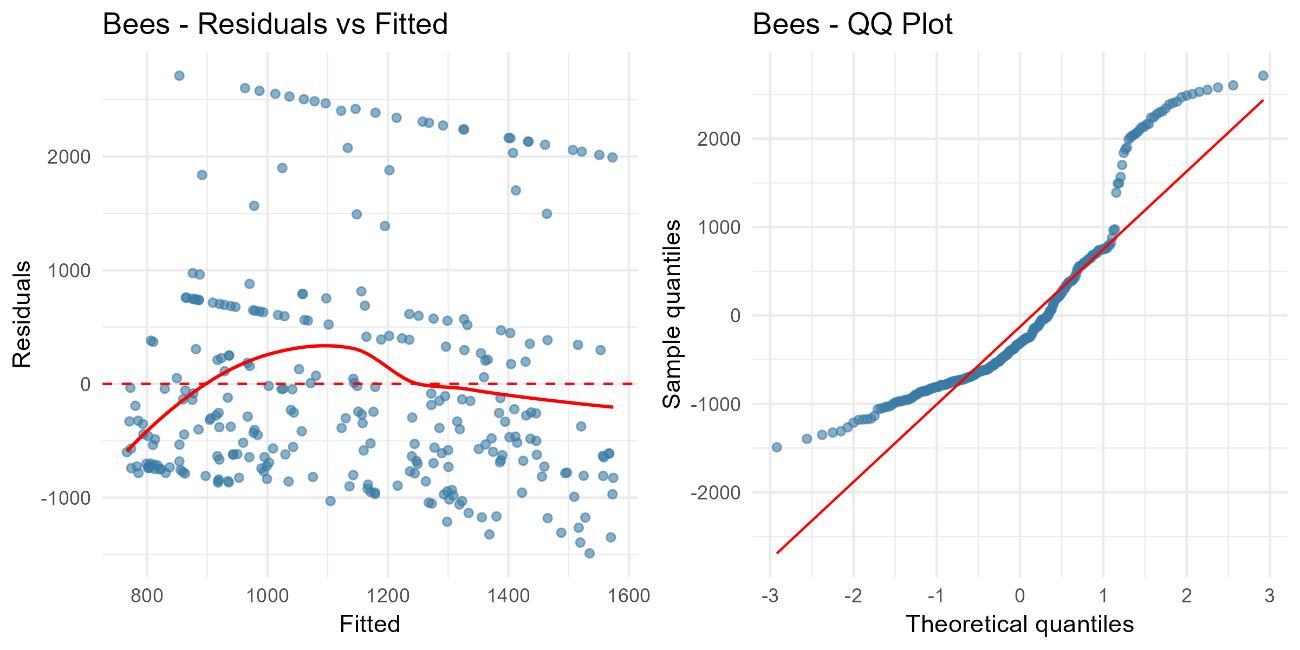
**

**
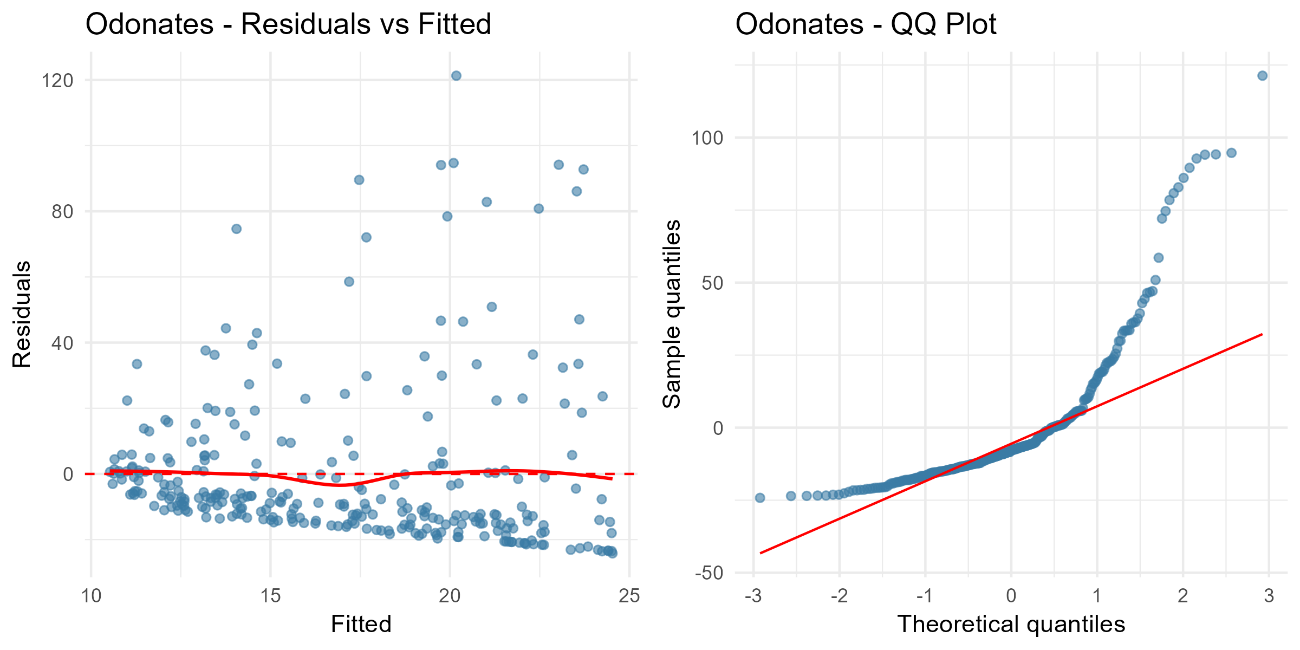
**

**
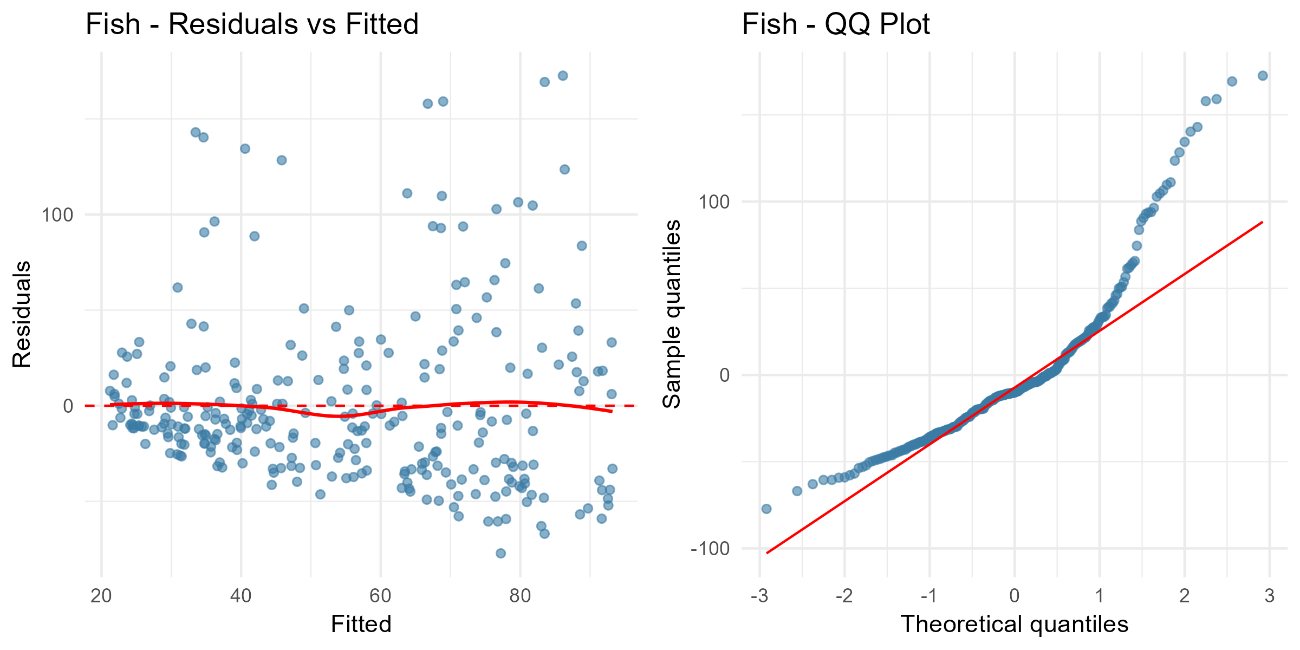
**

**
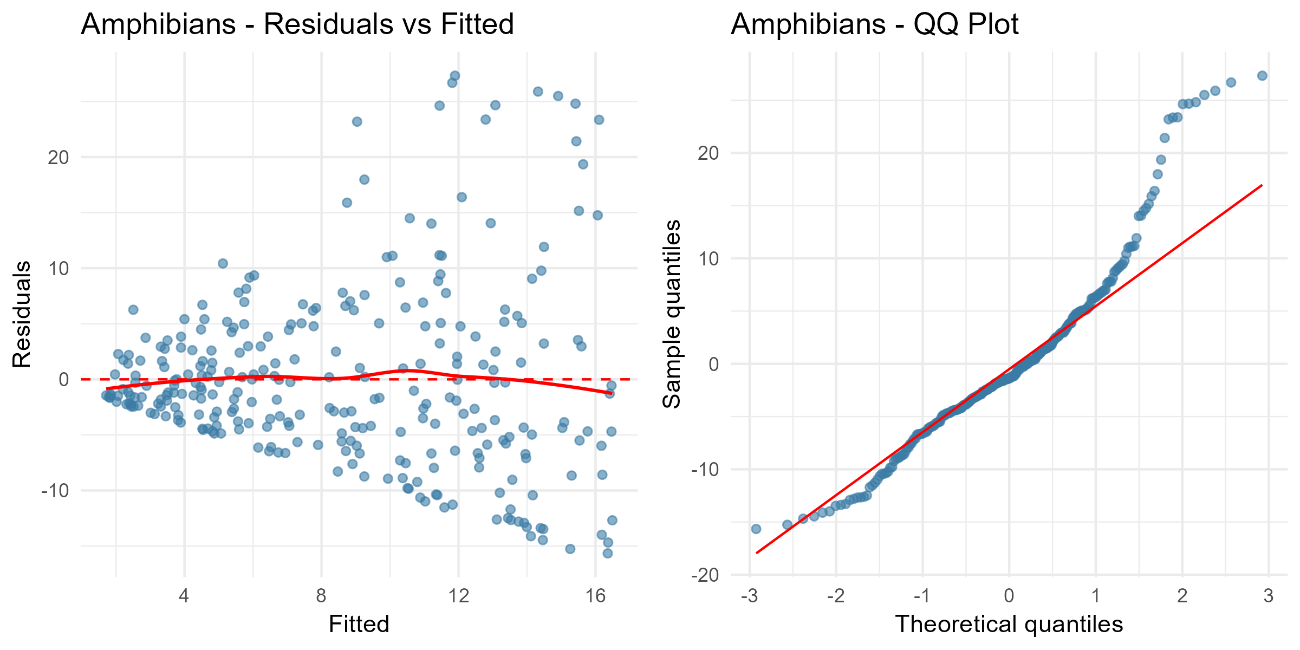
**

**
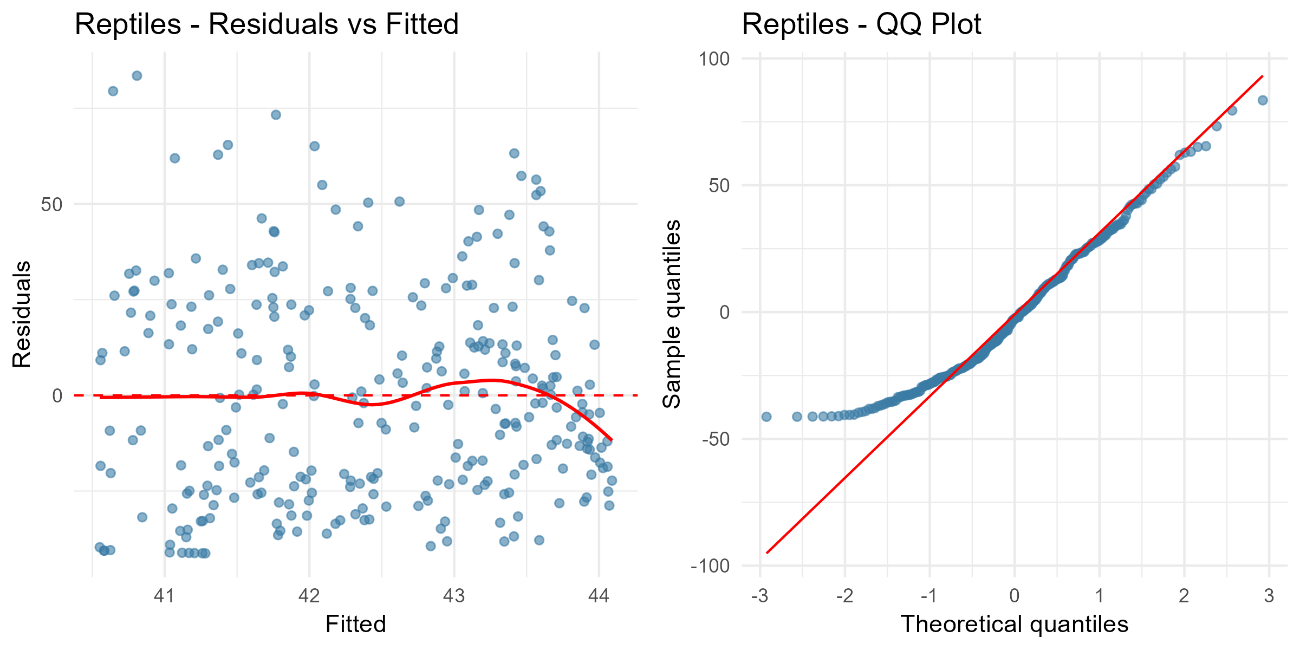
**

**
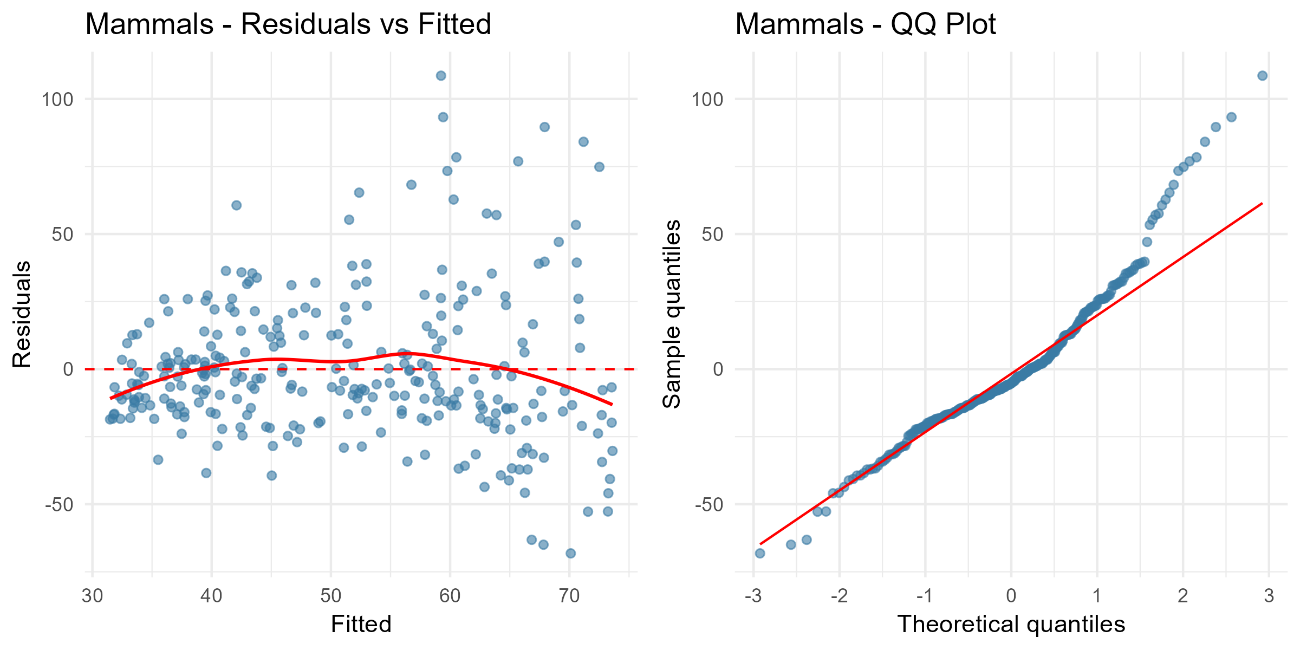
**

**
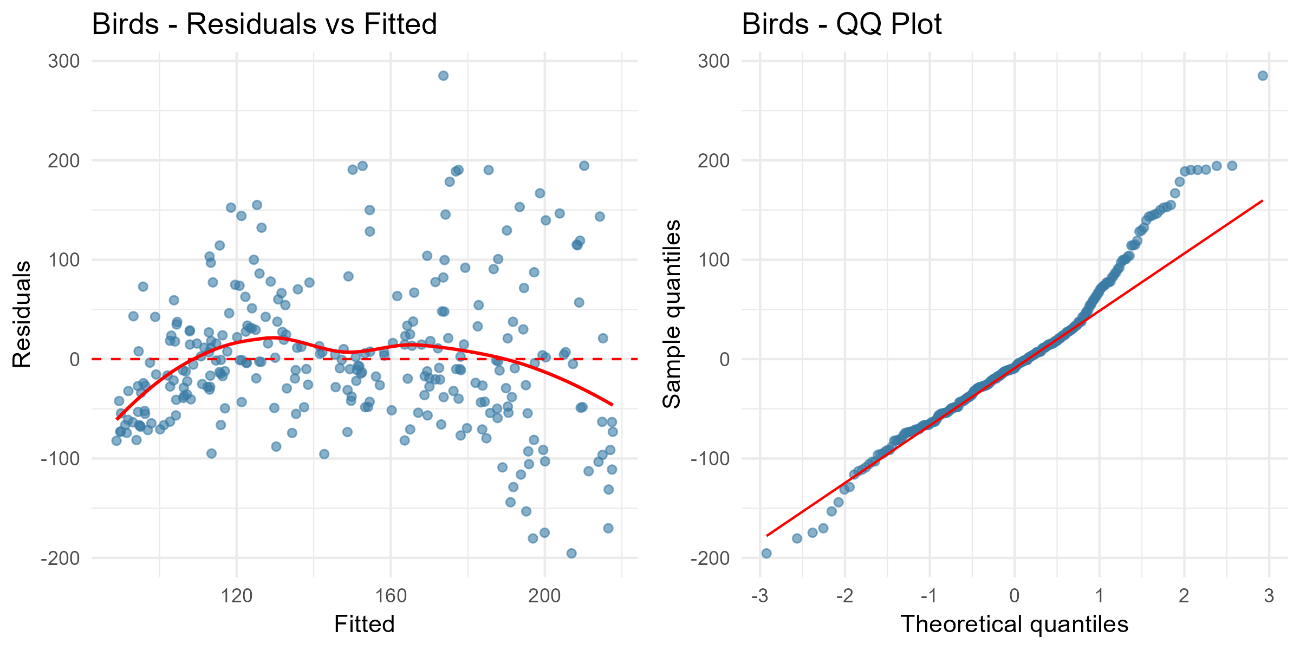
**

**
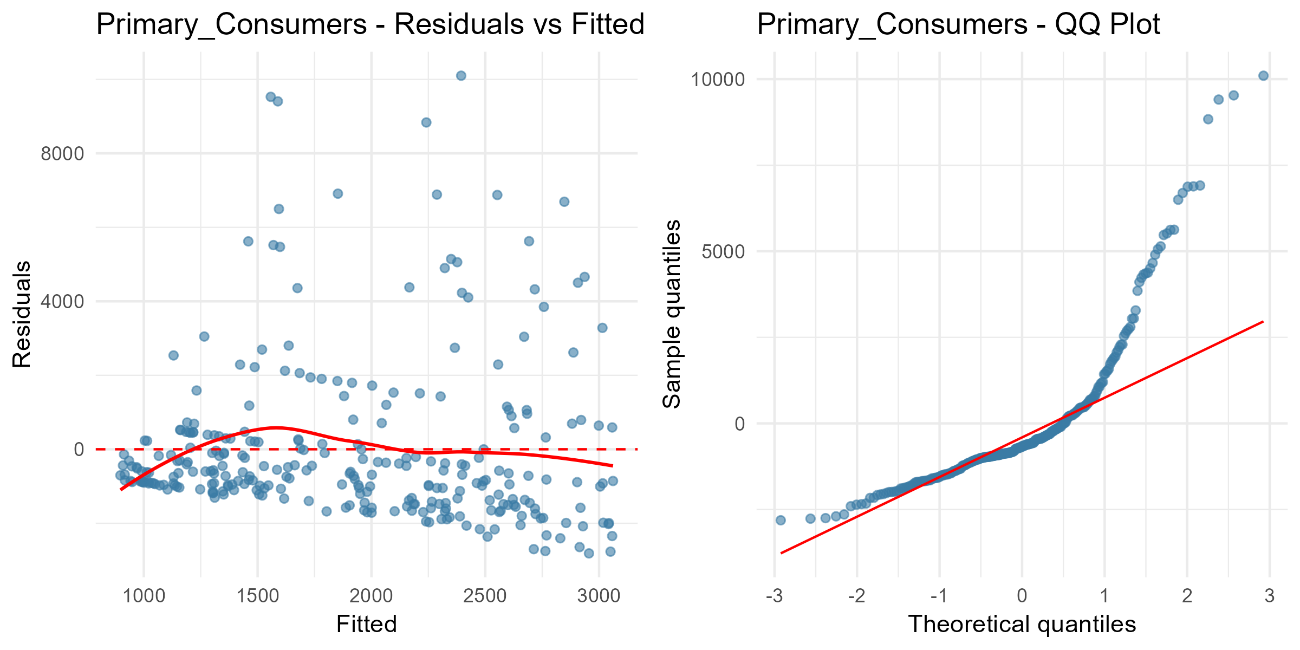
**

**
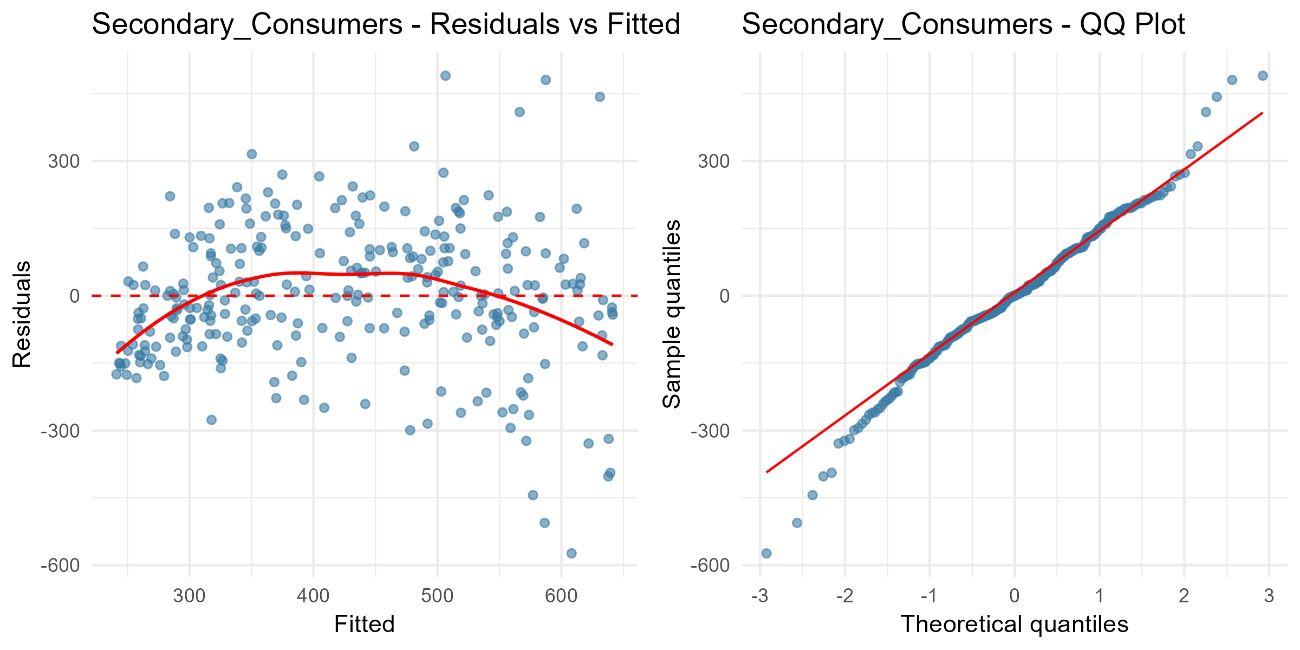
**

**
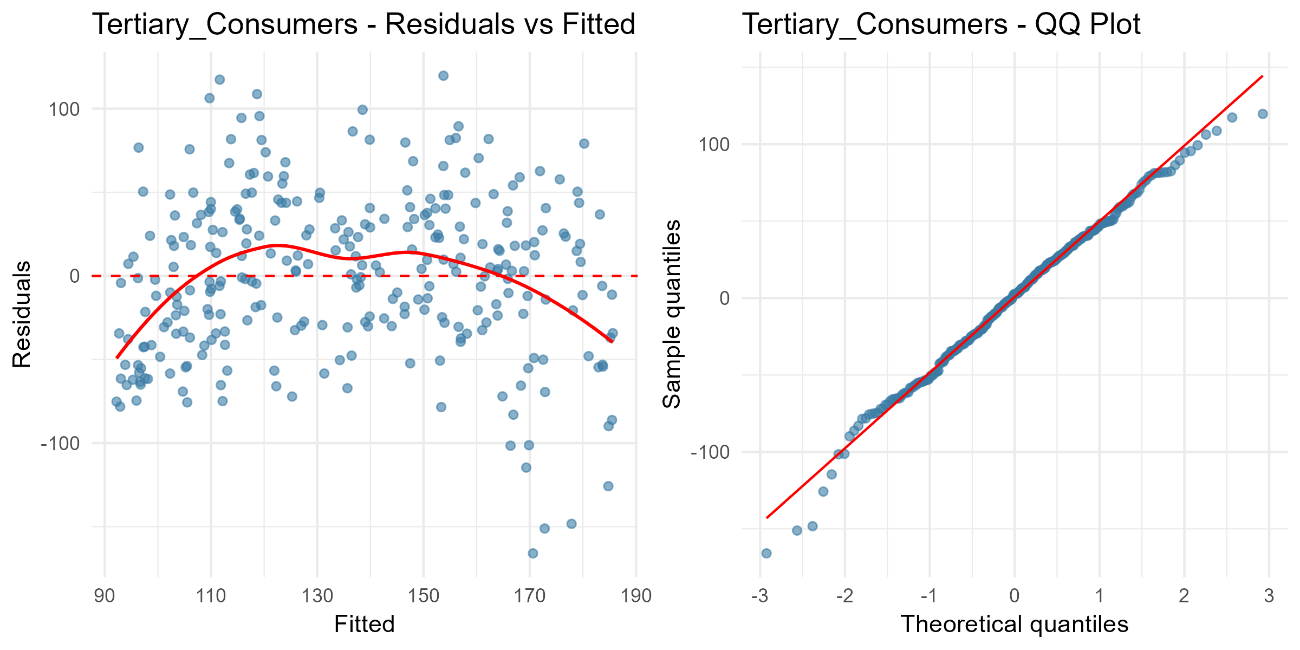
**

**
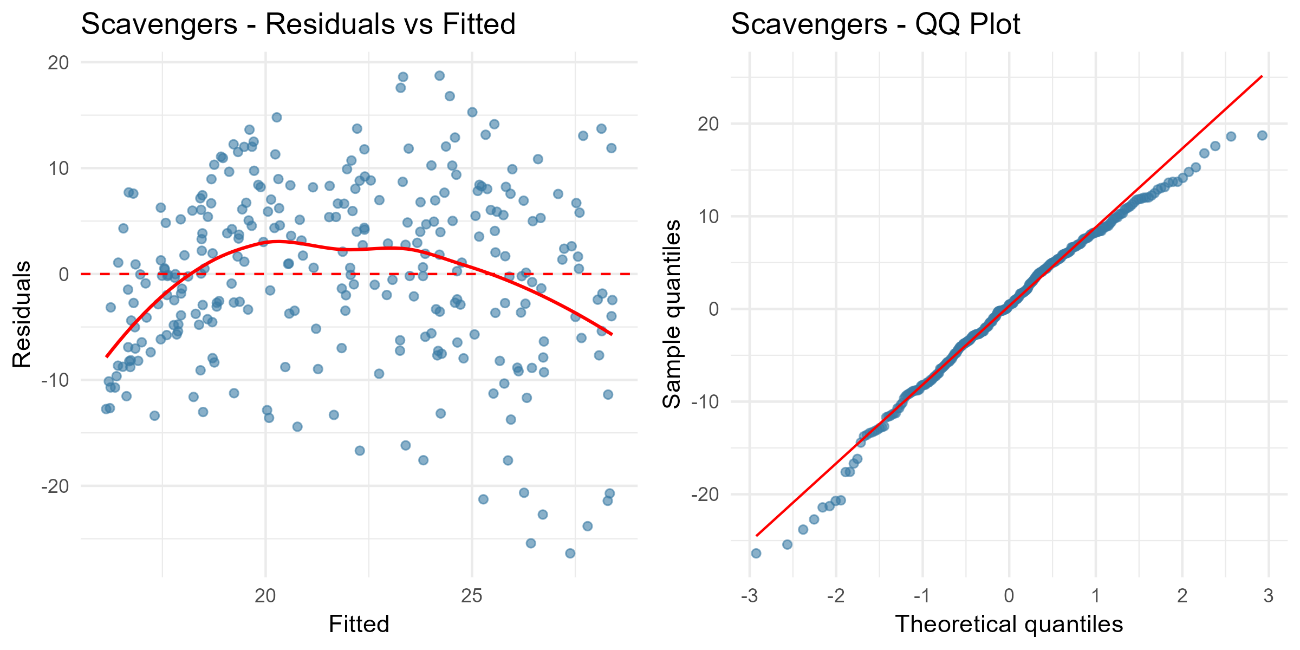
**

**
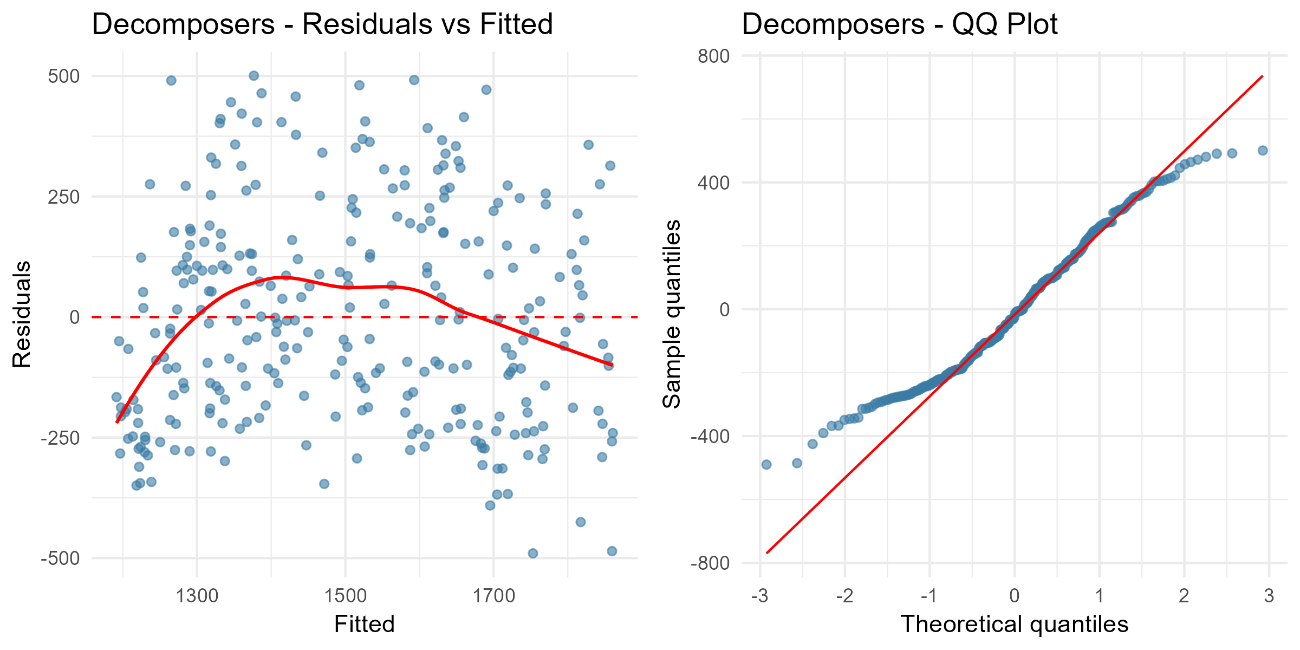
**

**Fig. S3.** Model diagnoses plots for aridity threshold models showing residuals vs fitted (left) and Q-Q plots (right) for each taxonomic and trophic group.

**
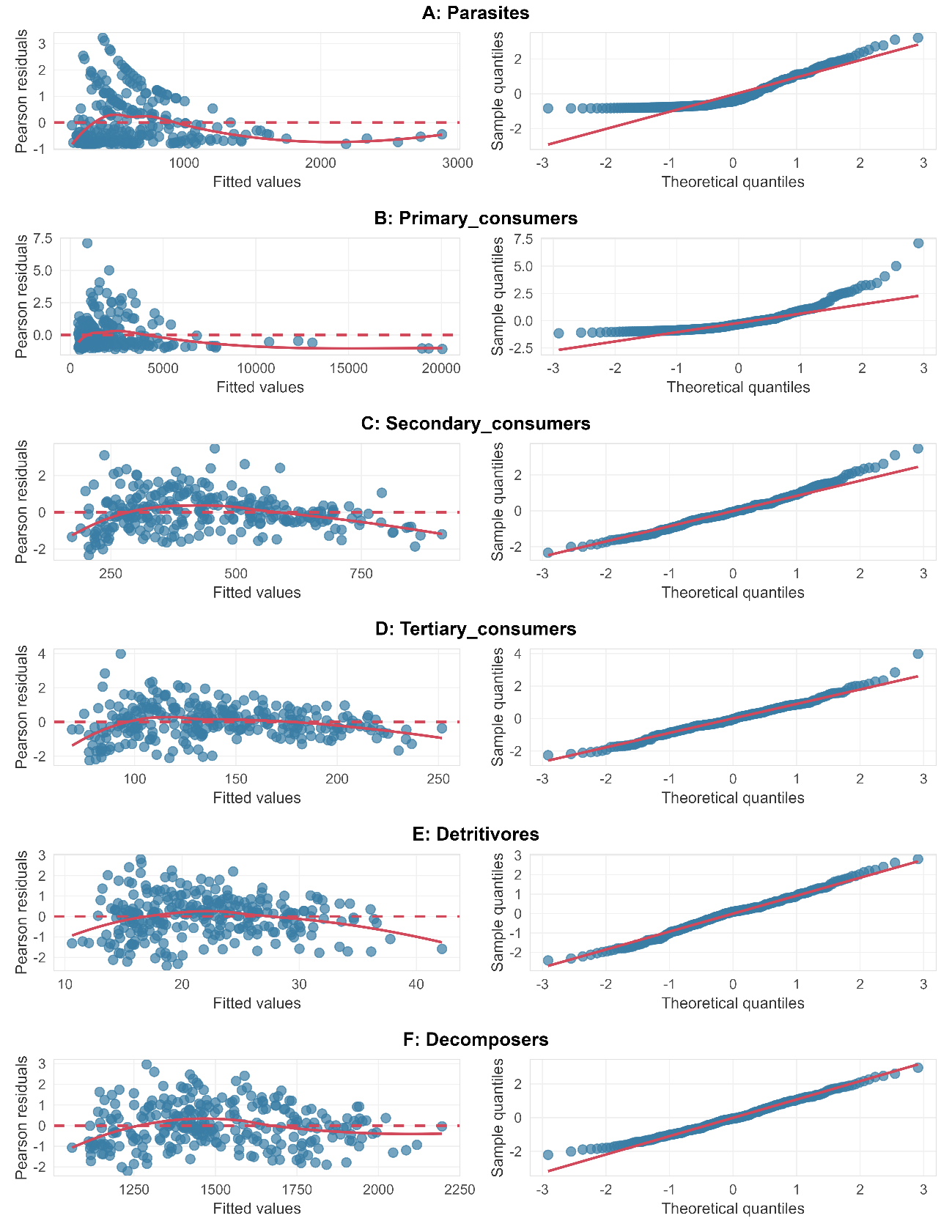
**

**Fig. S4.** Model diagnoses plots for Multitrophic richness models showing residuals vs fitted (left) and Q-Q plots (right) for each trophic group.

**REFERENCES**

Abell, R. et al. Freshwater ecoregions of the world: a new map of biogeographic units for freshwater biodiversity conservation. BioScience 58, 403–414 (2008).

Arderne, C., Zorn, C., Nicolas, C. & Koks, E. E. Predictive mapping of the global power system using open data. Sci. Data 7, 19 (2020).

Bartomeus, I., Stavert, J. R., Ward, D. & Aguado, O. Historical collections as a tool for assessing the global pollination crisis. Philos. Trans. R. Soc. B 374, 20170389 (2019).

Carlson, C. J., Dallas, T. A., Alexander, L. W., Phelan, A. L. & Phillips, A. J. What would it take to describe the global diversity of parasites?. Proc. R. Soc. B 287, 2020184 (2020).

CIESIN - Center for International Earth Science Information Network: Columbia University. Gridded Population of the World, Version 4 (GPWv4): Population density, Revision 11. NASA Socioeconomic Data and Applications Center (SEDAC). <https://doi.org/10.7927/H49C6VHW1> (2018).

Corbane, C., Florczyk, A., Pesaresi, M., Politis, P. & Syrris, V. GHS-BUILT R2018A - GHS built-up grid, derived from Landsat, multitemporal (1975-1990-2000-2014). European Commission, Joint Research Centre (JRC), [dataset]. <https://doi.org/10.2905/jrc-ghsl-10007> (2018).

Delgado-Baquerizo, M. et al. A global atlas of the dominant bacteria found in soil. Science 359, 320–325 (2018).

Falchi, F. et al. The new world atlas of artificial night sky brightness. Sci. Adv. 2, e1600377 (2016).

GBIF. GBIF occurrence download. doi: <https://doi.org/10.15468/dl.xh5y5g> (2020).

Guénard, B., Weiser, M., Gomez, K., Narula, N. & Economo, E.P. The Global Ant Biodiversity Informatics (GABI) database: synthesizing data on the geographic distributions of ant species. Myrmecological News 24, 83–89 (2017).

Gutiérrez‐Cánovas, C. et al. Large home range scavengers support higher rates of carcass removal. Funct. Ecol. 34, 1921–1932 (2020).

Jones, K. E. et al. PanTHERIA: a species‐level database of life history, ecology, and geography of extant and recently extinct mammals: Ecological Archives E090‐184. Ecology 90, 2648–2648 (2009).

Meijer, J. R., Huijbregts, M. A., Schotten, K. C. & Schipper, A. M. Global patterns of current and future road infrastructure. Environ. Res. Lett. 13, 064006 (2018).

Mikryukov, V. et al. Connecting the multiple dimensions of global soil fungal diversity. Sci. Adv. 9, eadj8016 (2023).

Nelson, A. et al. A suite of global accessibility indicators. Sci. Data 6, 266 (2019).

Phillips, H. R. et al. Global distribution of earthworm diversity. Science 366, 480–485 (2019).

Robinson, T. P. et al. Mapping the global distribution of livestock. PloS One 9, e96084 (2014).

Roll, U. et al. The global distribution of tetrapods reveals a need for targeted reptile conservation. Nat. Ecol. Evol. 1, 1677–1682 (2017).

RS-eco. *rasterSp: R Package to rasterize and summarise IUCN range maps*. R package version 0.0.1, commit 8c007900ceb2679f8aee04aec4d936648df5191b, <https://github.com/RS-eco/rasterSp> (2023).

Serna-Chavez, H.M. et al. Global drivers and patterns of microbial abundance in soil. Glob. Ecol. Biogeogr. 22, 1162–1172 (2013).

Tobias, J. A. et al. AVONET: morphological, ecological and geographical data for all birds. Ecol. Lett. 25, 581–597 (2022).
